# Tracing the human movements of three thousand years ago by volcanic grinding tools in the Final Bronze Age settlement of Monte Croce Guardia (Arcevia-Marche Region, central Italy)

**DOI:** 10.1038/s41598-023-34033-x

**Published:** 2023-04-29

**Authors:** P. Santi, A. Cardarelli, M. Bettelli, A. Di Renzoni, L. Cardarelli, C. Paniccia, A. Renzulli

**Affiliations:** 1 Dipartimento di Scienze Pure e Applicate, Università degli Studi di Urbino Carlo Bo, Urbino, Italy; 2 Dipartimento di Scienze dell’Antichità, Sapienza-Università di Roma, Rome, Italy; 3 Consiglio Nazionale delle Ricerche, Istituto di Scienze del Patrimonio Culturale (CNR-ISPC), Rome, Italy; 4 Dipartimento di Ricerca e Innovazione Umanistica, Università degli Studi di Bari Aldo Moro, Bari, Italy

**Keywords:** Environmental social sciences, Materials science

## Abstract

Volcanic rocks were among the most sought-after materials to produce grinding tools in antiquity because lavas lithologies, either mafic or felsic, ensured good wear resistance and grinding capacity with respect to many other kinds of rocks. The interest in findings made of vesciculated lavas, referable to parts of querns, mortars, and/or pestles of the Final Bronze Age site of Monte Croce Guardia (Arcevia) lies in the fact that this settlement was built upon limestones belonging to the sedimentary sequence of the Marche-Umbria Apennines (central Italy) and far away from potential raw materials of volcanic rocks. A petrologic study of 23 grinding tool fragments clearly indicates a provenance from the volcanic provinces of central Italy: Latium and Tuscany Regions. Few leucite tephrites (5) and one leucite phonolite lavas have a clear magmatic affinity with the high-K series of the Roman Volcanic Province (Latium) whereas the most abundant volcanic lithotype (17 samples) is represented by shoshonites (K-series) whose thin section texture, modal mineralogy and major-trace elements contents closely match with the shoshonite lavas from the Radicofani volcanic centre in the Tuscan Magmatic Province. At Radicofani (a volcanic neck in the eastern sector of Tuscany) a Final Bronze Age site coeval to that of Arcevia is present and a potential pathway corridor from that site towards Arcevia (air-line distance of ca. 115 km) is dotted with many settlements of the same age. Through analytical algorithms based on the slope and the different human-dependent cost-functions which can be applied to determine non-isotropic accumulated cost surface, least-cost paths and least-cost corridors, the best route from Radicofani to Monte Croce Guardia, approximately 140 km long, was simulated, with a walking time of 25–30 h, possibly using pack animals and wheel chariots. Three thousand years ago the Apennine Mountains did not thus constitute a barrier for human movements. This study also shed light on some other possible patterns of interactions between Final Bronze Age communities of central Italy through the present-day regions of Tuscany, Umbria and Marche, aimed towards the best performance of strategic economic activities at that time such as that of the transformation of cereals, and accompanied to cultural and social reasons.

## Introduction

**Figure 1:**
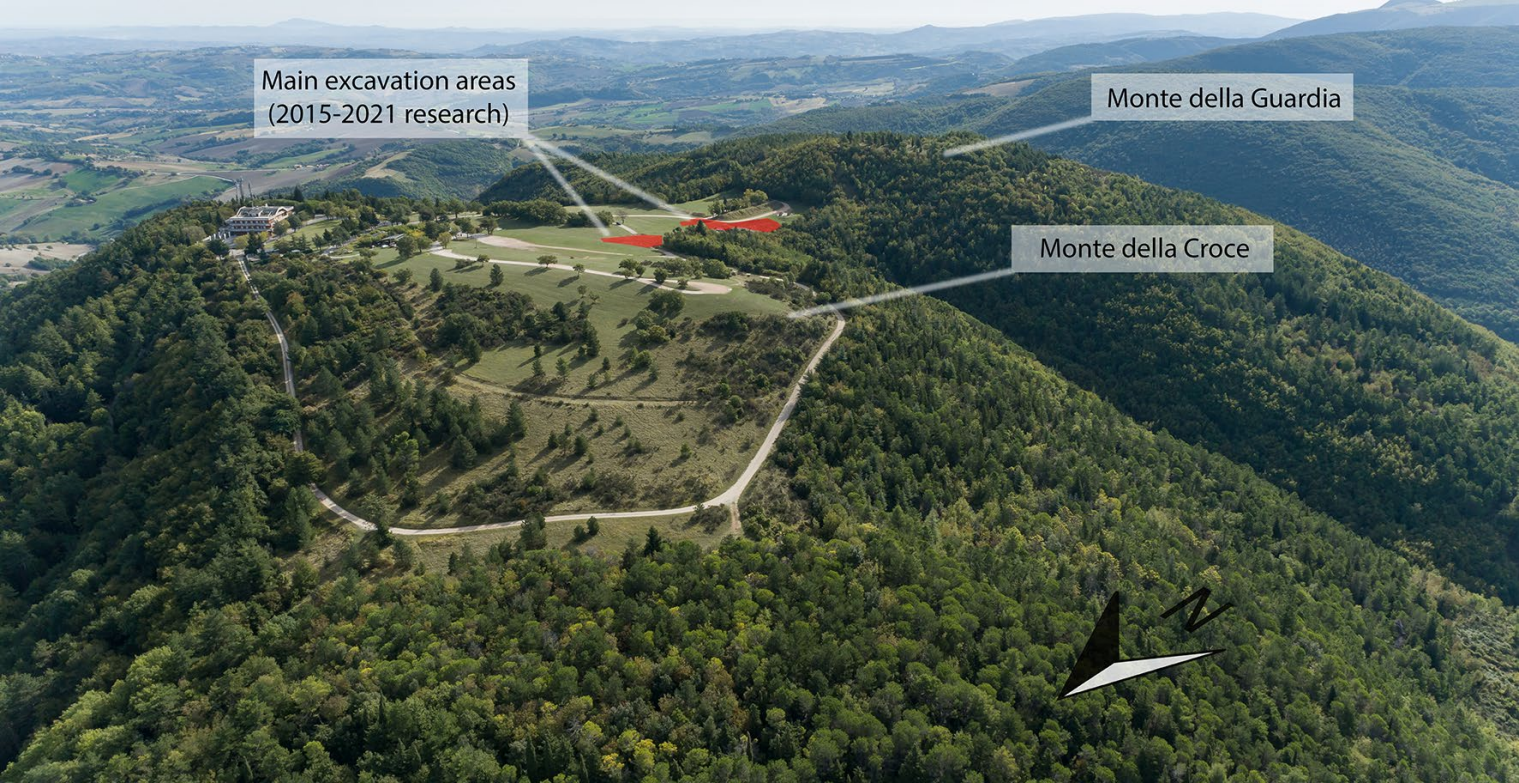
Drone aerial view of the Monte Croce Guardia archaeological site, with the indication of the main research areas of the 2015–2021 campaigns (photo by A. Conte).

**Figure 2:**
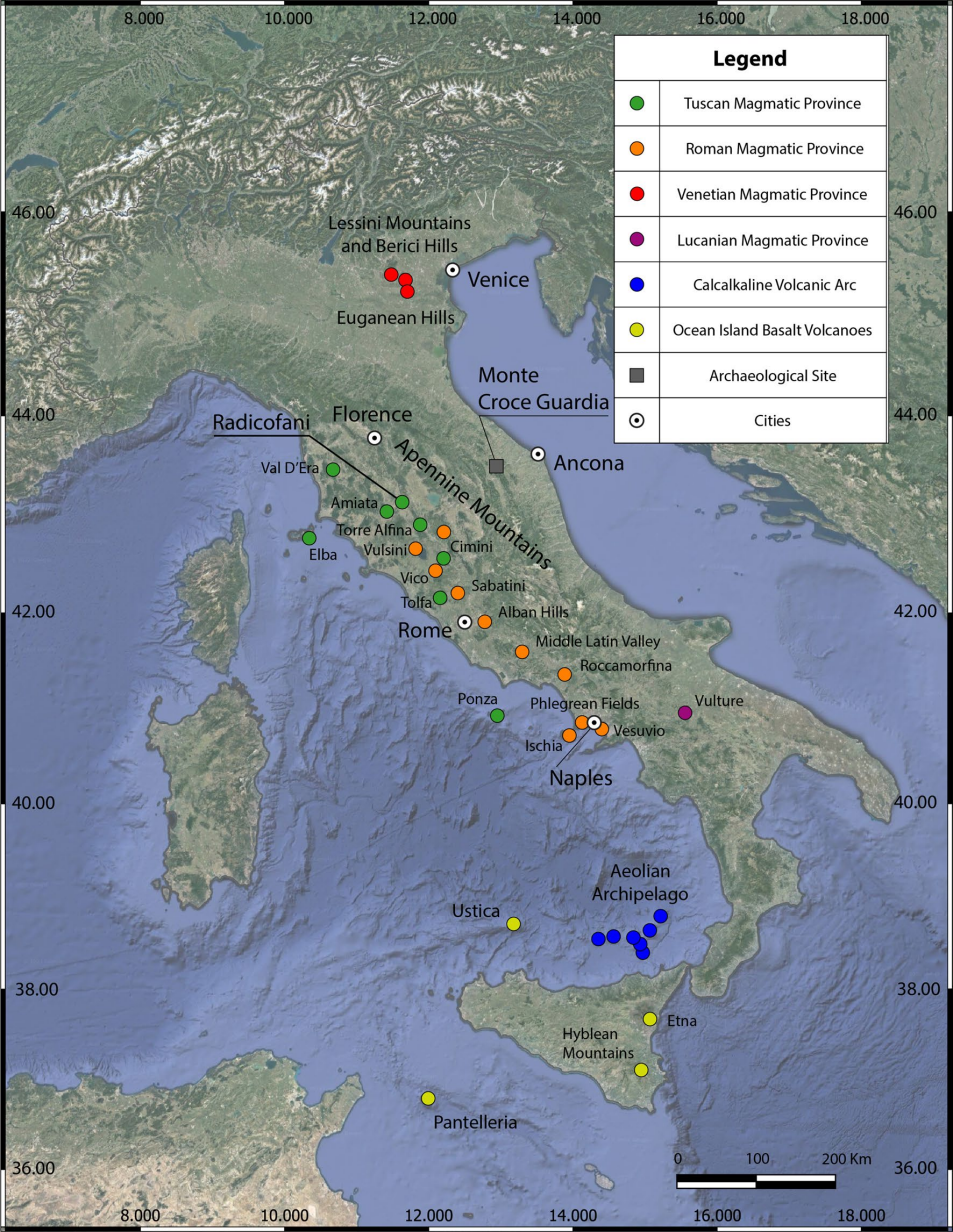
Distribution of the main volcanism in Italy with related magmatic provinces/volcanoes (modified)^3^ and the location of the Monte Croce Guardia archaeological site.

The site of Monte Croce Guardia was settled during the advanced phase of Recent Bronze Age (first half of twelfth century BCE) and flourished in the Final Bronze Age (1150-925 BCE), when it was densely inhabited. It is located on a dominant and well defended hilltop, with an excellent visual control of the surrounding landscape. The occupied area can be estimated between 22 and 27 hectares, corresponding to the almost flat surface that characterises the summit of Monte della Croce, Monte della Guardia and the saddle connecting the two mountaintops (Fig. 1). The site has been long investigated by Superintendence of the Marche Region since the ’60 of last century, when it was discovered, till ’70. Another campaign took place in 1995. A new and still ongoing cycle of research, directed by one of the authors (A.C.), started in 2015. This latest research has highlighted the existence of numerous houses with a rectangular plan, many of which exceed 100 m^2^ (Fig. S1). According to a rough approximate, the number of houses and annexed structures can be estimated at between 80 and 100 units^1,2^. The archaeological discoveries revealed the existence of specialized workshops for metal-working and other handicraft products. Furthermore, the presence of “exotic” materials, such as amber and glass ornaments, testifies the relevance of the settlement and that it was part of a long-distance exchanges network, also including grinding tools (millstones and/or pestles) as evidenced by the stone fragments investigated in the present work. These latter were, of course, imported from far away volcanic source areas as the Monte Croce Guardia settlement was established upon the limestones belonging to the sedimentary sequence of the Umbria-Marche Apennines, widely present within a ratio of several tens of kilometres from Arcevia. A petrological comparison between 23 fragments of grinding tools of Monte Croce Guardia and the lavas belonging to the main volcanic provinces of central-northern Italy (Fig. 2) will be performed, according to compatibility on thin section modal mineralogy and textures, major and trace elements, and their magmatic series. Using petrographic and chemical comparisons we are going to define volcanic provenance areas of the Monte Croce Guardia grinding tools and to trace the millstones trade networks that accompanied the human interactions, from the original rock sources to the final destination (present archaeological site). Following common archaeological methods to detect prehistoric pathways and routes^4–8^, least-cost and cost-surface analyses^9–12^ will allow analytical algorithms to estimate and simulate the human movements, starting from the recognized volcanic source areas of provenance of the investigated grinding tools.

### Volcanic millstone trade in antiquity

Volcanic rocks (mainly lavas) are recognized in antiquity as highly prized geomaterials for millstones, mortars, and pestles, because of very high-performance in grinding cereals, acorns, or fruits. Good wear resistance, abrasive ability and rough vesicular surfaces are the physical properties that give to the lavas the best quality of rock for grinding stones^13–20^. The technological history of the volcanic millstones started from saddle querns, very widespread from the Upper Palaeolithic^13,16,19,21^ up to the Bronze-Iron Age^22^ and the Phoenician-Punic period^23^. Mortars and saddle querns were indeed the first tools used by humans for grinding a wide range of products. Current archaeological data points to a use of querns for grinding cereals in the Near East (Israel; flat basalt stone) as early as the Upper Paleolithic (ca. 20,000 yr. BCE), i.e., 10,000 yr. before cereal domestication^24^. The increasing demand for flour, necessary to meet the bread-making needs of the growing population in the Mediterranean area^20^ lead to more efficient grinding devices such as the hopper-rubber millstones (starting from the fifth century BCE, Olinthian-type^25–27^) small to medium size rotary devices (e.g., Morgantina-type, from the fourth-third century BCE^15,25,28–31^) and finally large hourglass rotary millstone (Pompeian-type; from the third-second century BCE^16,25,32–35^).

Literature data on the provenance of the lava millstones from the Protohistoric period to the Roman Empire are abundant. Most researches point out the italian volcanic areas of provenance^36^: the Roman Volcanic Province such as the Vulsini Volcanic Complex with a strongly exploited leucite phonolite quarrying site near Orvieto^17,37–39^ and Vesuvius^40,41^; Vulture Volcano^42–44^; eastern Sicily volcanoes: Etna and Hyblaean Mountains^26,31,39,44–47^; Sardinia volcanic province, mainly the Mulargia site^44,48^; volcanic islands of the Sicilian Channel, mainly Pantelleria^23,49^ or the southern Tyrrhenian Sea such as those of the Aeolian Archipelago and Ustica Island^46,50,51^; volcanic islands of the Aegean Sea^15,30,44,52,53^. In addition, millstones found in shipwrecked cargoes contributed to exactly trace the trade networks from the volcano/volcanic area of provenance to the final destinations^23,30,36,54^. By contrast, literature data on the provenance of the volcanic grinding tools used and transported during the Bronze-Iron Age is less abundant^20,22,43,45,50,55–59^. Some volcanic grinding tools (or fragments of them) found in the archaeological site of Frattesina di Fratta Polesine (Rovigo, Veneto Region), dated between eleventh and eighth century BCE^57^, are mainly represented by trachytes from the Euganean Hills and some leucite-bearing lavas from the Roman Volcanic Province (inferred to come from the leucite phonolite quarries near Orvieto).

## Sampling and methods

**Figure 3:**
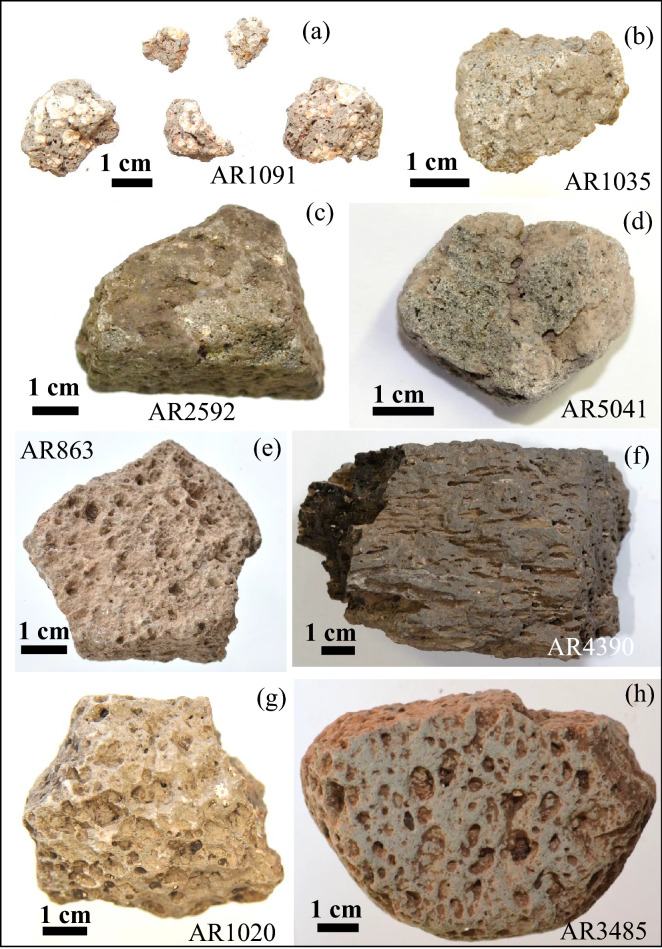
Macroscopic view of representative investigated fragments referable to stone grinding tools. (**a**) leucite phonolite; (**b**–**d**) leucite tephrites; (**e**–**h**) shoshonites.

Sampling in the archaeological site of Monte Croce Guardia (Arcevia) was performed on 23 fragments of volcanic grinding tools (Table S1; Fig. 3). A maximum volume of two cubic centimetres was removed from the original samples for thin sections and chemical analyses. The volcanic samples can be referred to grinding tools, based on their shape and other physical features (e.g., smooth surfaces). In particular, they could be considered fragments of either querns (base- or hand-stone) or mortars. The studied artifacts consist of fragments of grey, dark grey or black scoriaceous lavas with different degree of porphyricity and vesicularity (Fig. 3). In some of them the polished (smooth) surface due to the use as grinding tools is still clearly visible (e.g., Fig. 3h). The investigation of these volcanic samples to establish the provenance was carried out based on the fundamental igneous petrology methodology to define classification and magmatic series (Table S1). Thin section modal mineralogy and petrographic texture of the 23 samples were determined through a polarized light optical microscopy. Whole-rock chemistry was determined at the Activation Laboratories LTD (Ancaster, Canada) by ICP-OES (Inductively Coupled Plasma-Optical Emission Spectrometry; Varian Vista 735) and ICP-MS (Inductively Coupled Plasma-Mass Spectrometry; Perkin Elmer Elan 9000) for major (wt%) and trace elements (ppm) respectively. Samples were crushed and powdered in an agate mortar to avoid contamination as much as possible and fused by lithium metaborate/tetraborate technique in an induction furnace, providing a fast and high-quality fusion. The resulting molten bead was rapidly digested in a weak (5%) nitric acid solution containing an internal standard and mixed continuously until completely dissolved. It is only with this attack that major oxides including SiO_2_, refractory minerals (i.e., zircon, sphene, chromite, etc.), REE and other high field strength elements are put into solution. Calibration was performed using 14 prepared USGS and CANMET certified reference materials. One of the 14 standards is used during the analysis for every group of ten samples. Errors, calculated using the certified natural rock standards and replicates of some samples, are generally < 2% and < 5% for major oxides and trace elements respectively. Detection limit for each analysed element is shown in Table S2.

Concerning the testing on topographic connectivity between the provenance areas and the final destinations of volcanic grinding tools, DEM and the other geographic features were handled with QGIS 3.28 Firenze. The same software was used to create the maps in the text. Cost-surface and least-cost path analyses were performed with the Movecost (1.8) package within the R environment (4.1.2). The graphs are carried out with the seaborn library (0.11.2) available for the Python programming language (3.9.15). The entire processing took place on a i7-12700 K processor PC.

## Results

### Major oxides, chemical classification and petrographic characterization

**Figure 4:**
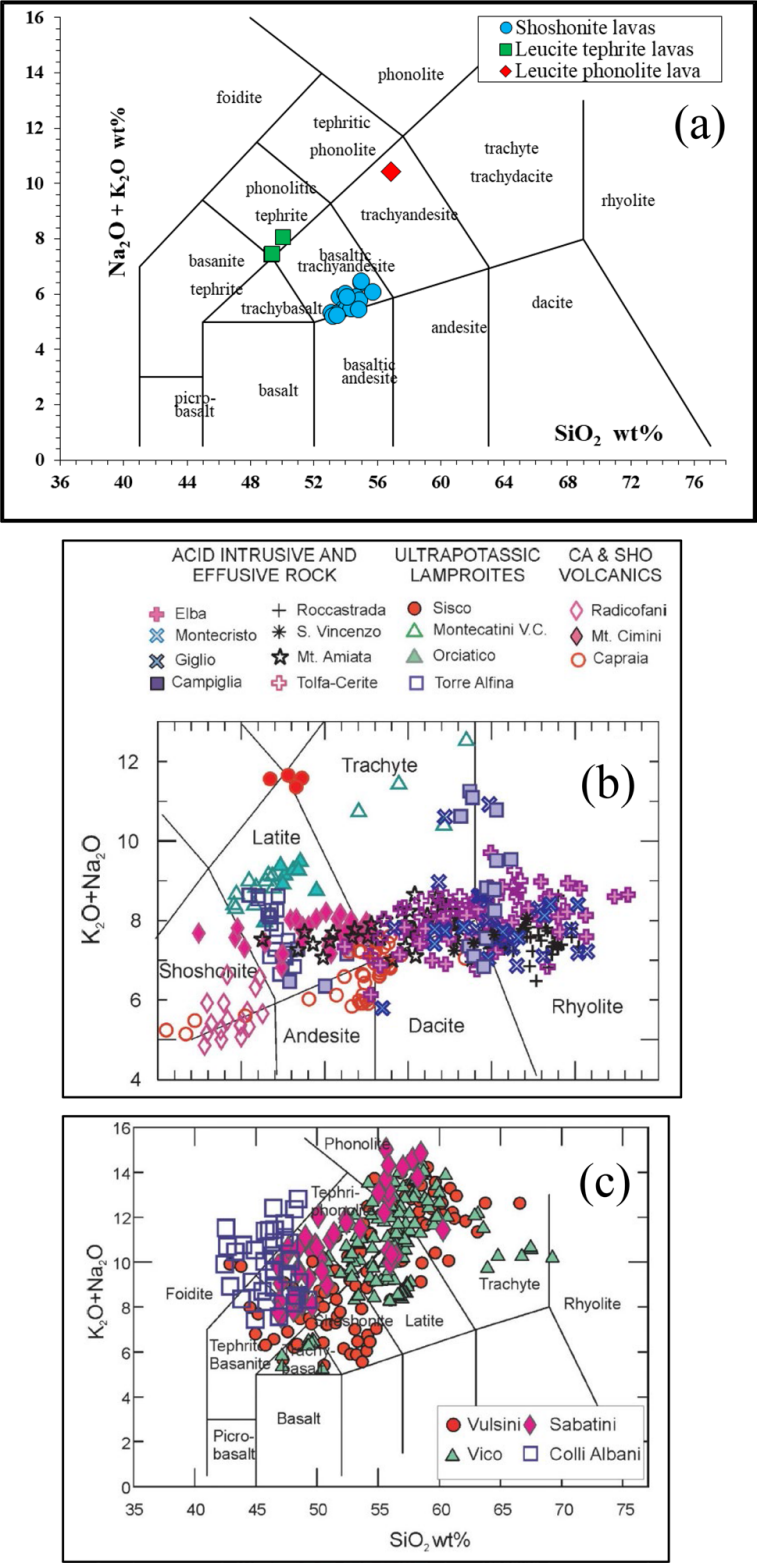
The Total Alkali—Silica (TAS)^60^ classification diagrams for the fragments of volcanic grinding tools from Monte Croce Guardia (**a**), the whole rocks of the Tuscan Magmatic Province^61^ (**b**) and the products of the four main areas (central-northern Latium) of the Roman Volcanic Province^61^.

Some of the studied volcanic grinding tools are characterized by Loss of Ignition (LOI) values > 3.0 wt% (Table S2) indicating the presence of some secondary phases or pseudomorphic minerals, partly affecting the original major and trace elements composition of the samples. To avoid the use of erroneous petrographic terms, the two samples with the highest LOI values (AR2592: 7.06 wt%; AR1981: 7.35 wt%) were not plotted both (1) in the chemical Total Alkalis (Na_2_O + K_2_O) vs. Silica (SiO_2_) diagram (TAS, Fig. 4a) recommended by the IUGS^62^ for classification of the volcanic rocks and (2) in any chemical diagrams of the present paper. The use of major elements on anhydrous basis in the TAS diagram (i.e., abundances recalculated at 100 wt% basis with proportional redistribution of the LOI wt% to each major oxides) should mitigate the effect of misleading classification for medium high-LOI (3.09 and 5.33 wt%) samples (AR5041, AR1035, AR1091 and AR679). However, for all the samples with LOI > 3.0 wt% the use of a thin section classification (QAPF^63^) by modal mineralogy (instead of TAS) seems to be more appropriate. Using the TAS diagram (Fig. 4a) we can correctly identify the most abundant samples (17) with relatively low LOI (0.78–2.58 wt%) as shoshonites (i.e., basaltic trachyandesites with Na_2_O – 2.00 ≤ K_2_O) therefore belonging to a magmatic K-series (Table S1). On thin section, these shoshonite samples are characterized by lavas with Porphyritic Index (P.I.) ranging from 5 to 20 vol% and a vesiculation of 15–30 vol% (Fig. 3 and Fig. S2a–d). The phenocrysts are represented by well-developed euhedral to subhedral olivine (partially altered to bowlingite and iddingsite) and clinopyroxene. The groundmass is microcrystalline (intergranular texture) to glassy-cryptocrystalline, mainly consisting of plagioclase laths >  > olivine ≥ clinopyroxene. Opaque minerals (magnetite) are the main accessory phases. Besides the clear classification in the TAS diagram, all these shoshonite samples show homogeneous Al_2_O_3_ (14.3–15.9 wt%), Fe_2_O_3tot_ (6.4–7.5 wt%), MgO (7.5–8.9 wt%), CaO (6.6–8.4 wt%) and TiO_2_ (1.0–1.2 wt%).

Among the samples with LOI values > 3.0 wt%, thin section modal mineralogy of five of them (AR679, AR1035, AR1981, AR2592, AR5041) are very similar and therefore can be grouped as belonging to the same lithotype. They are represented by mostly aphyric lavas (P.I. ≤ 5 vol%) with vesiculation up to 15 vol% (Fig. S2e–g). Phenocrysts and microphenocrysts of leucite, zoned clinopyroxene with green cores and colourless rims and plagioclase are the fundamental mineral assemblage. Groundmass is microcrystalline (intergranular) with some cryptocrystalline domains and shows a general alteration to secondary minerals also filling the vesicles. Magnetite is the main accessory phase. On the basis of modal mineralogy, this petrographic group can be classified as leucite tephrites (High K-series; Table S1) although they fall just outside the tephrite field in the TAS. Chemical analyses of these leucite tephrites show a relatively homogeneous composition for Al_2_O_3_ (16.4–17.8 wt%), Fe_2_O_3tot_ (7.6–8.7 wt%), MgO (3.9–4.8 wt%), CaO (9.7–11.3 wt%) and TiO_2_ (0.7–0.9 wt%).

Finally, the sample AR1091 (LOI 4.93 wt%) is characterized by a vesicularity of 5–10 vol% (Fig. S2h) and a modal mineralogy, both as phenocrysts (P.I. ca. 15 vol.%) microphenocrysts and groundmass (microcrystalline with a pilotassitic texture), consisting of leucite, plagioclase (always rimmed by low Ca-feldspar), sanidine and green clinopyroxene (often with opaque inclusions). Some glomerophyres, constituted by the same phases described as phenocrysts and groundmass, are also present. Accessory minerals are mainly represented by opaque minerals (magnetite). On the basis of the above modal mineralogy, a classification as a leucite phonolite (High K-series; Table S1) is the most appropriate for this sample, although loss of alkalis due to secondary alteration prevent the proper term in the TAS diagram (Fig. 4a). As a matter of fact, all the other oxides abundances (Al_2_O_3_ 20.4 wt%; Fe_2_O_3tot_ 4.2 wt%; MgO 0.7 wt%; CaO 4.4 wt%; TiO_2_ 0.6 wt%) are typical of phonolite rocks.

### Trace elements

**Figure 5:**
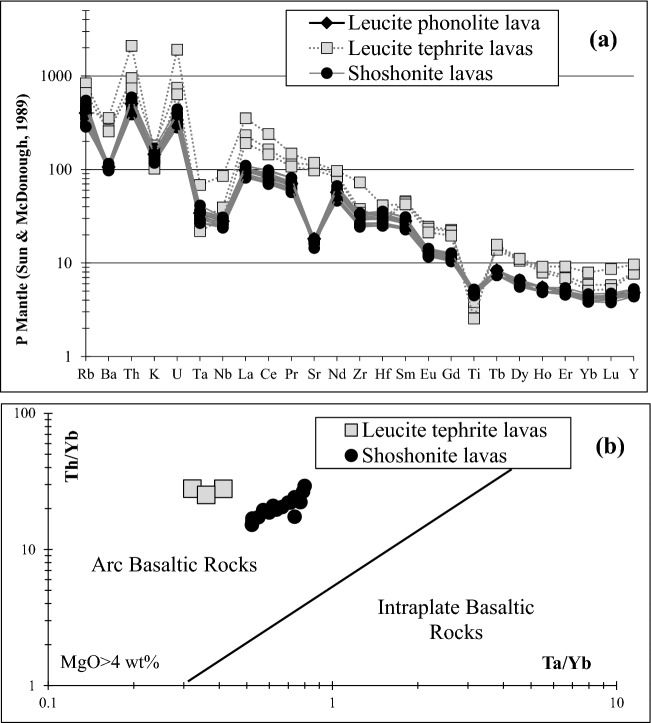
(**a**) Incompatible trace elements patterns normalized to Primitive Mantle^64^; (**b**) Th/Yb vs Ta/Yb discriminant diagram^67^.

According to the patterns of the incompatible trace elements, normalized to Primitive Mantle, reported in Fig. 5a, all the 23 samples are characterized by the Ta-Nb-Ti negative anomalies of subduction related volcanic rocks^65,66^. The patterns are very similar (sub-parallel) for all the samples, except for Sr-negative anomalies for the shoshonites. In addition, the leucite phonolite shows the enrichment, with respect to the other samples, of most of the incompatible trace elements due to the origin from a more evolved magma. The subduction-related Volcanic Arc fingerprint of all the samples can be also emphasized in the Th/Yb vs. Ta/Yb (Fig. 5b) discriminant diagram for relatively undifferentiated (basaltic, MgO > 4 wt%) rocks, where an intraplate origin is clearly ruled out for all the grinding stones. All the shoshonite samples are relatively homogeneous for compatible elements (Cr 470–550 ppm, Co 30–33 ppm, Ni 180–260 ppm), LREEs (La 56–80 ppm; Ce 124–187 ppm, Nd 63–101 ppm) and HFSEs (Nb 17–23 ppm, Th 33–58 ppm, U 6–10 ppm) whereas LILEs, being relatively mobile during secondary alteration, show a wider compositional range (Rb 182–352 ppm, Ba 682–809 ppm) excepting for Sr (305–387 ppm). Concerning the trace elements compositional range of the leucite tephrite grinding stones (excluding the two samples with LOI > 7.0 wt% where the trace elements abundance could have been strongly affected by secondary alteration) the enrichment of LREEs (La 132–160 ppm, Ce 258–292 ppm, Nd 113–118 ppm), LILEs (Rb 418–531 ppm, Ba 1786–1845 ppm, Sr 2071–2484 ppm) and HFSEs (Nb 21–28 ppm, Th 63–81 ppm, U 13–16 ppm, Zr 387–449 ppm) is typical of the High K-series (with respect to the K-series at the same degree of evolution^68^). Also, the leucite phonolite emphasizes a trace elements distribution closely matching a High K-series^68^, with an extreme enrichment in incompatible elements such as La (243 ppm), Ce (427 ppm), Nb (61 ppm), Ba (2493 ppm), Sr (2489 ppm), Th (179 ppm) and U (40 ppm).

## Discussion

### Petrological constraints

Following igneous petrology, when trying to establish the provenance of volcanic grinding tools used in antiquity in the Mediterranean area, the starting procedure should be defining not only the rock-name but also the magmatic series affinity of the stone artifact. In fact, the magmatic series of the volcanic rocks characterizing the whole Mediterranean region, are strictly linked to the geodynamics (Plate Tectonics) and relative mantle melting processes and can be roughly distinguished in two general groups, namely (1) anorogenic^69^ and (2) subduction-related^3,70–73^ series. All the volcanic rocks of central Italy (Fig. 2) belong to the subduction-related group^68,72^ namely the Tuscan Magmatic Province (intrusives and extrusives from Tuscany to northern Latium) and the Roman Volcanic Province (extrusives from northern Latium to northern Campania, including Ernici and Roccamonfina). Few volcanic rocks in Umbria are also linked to the subduction-related geodynamics of central Apennines^68^. Moreover, concerning the volcanoes of the Neapolitan area (Somma-Vesuvius, Phlegrean Fields and Ischia Island) and the Aeolian Archipelago, also belonging to an Arc volcanism (Fig. 2), it seems to be not reasonable to consider them as candidate provenance area, due to very high distances. The anorogenic (intraplate, mainly Na-series^3,68,69^; Fig. 2) volcanic rocks of the Veneto Magmatic Province in the NE Italy (i.e., Euganean Hills, Berici and Lessini Mountains^74,75^) and Sicily (e.g., Etna, Hyblaean Mountains^69^) or Sicilian Channel (e.g., Pantelleria Island^76–78^) are all ruled out as provenance, for their incompatible magmatic series fingerprint with the investigated grinding tools.

**Figure 6:**
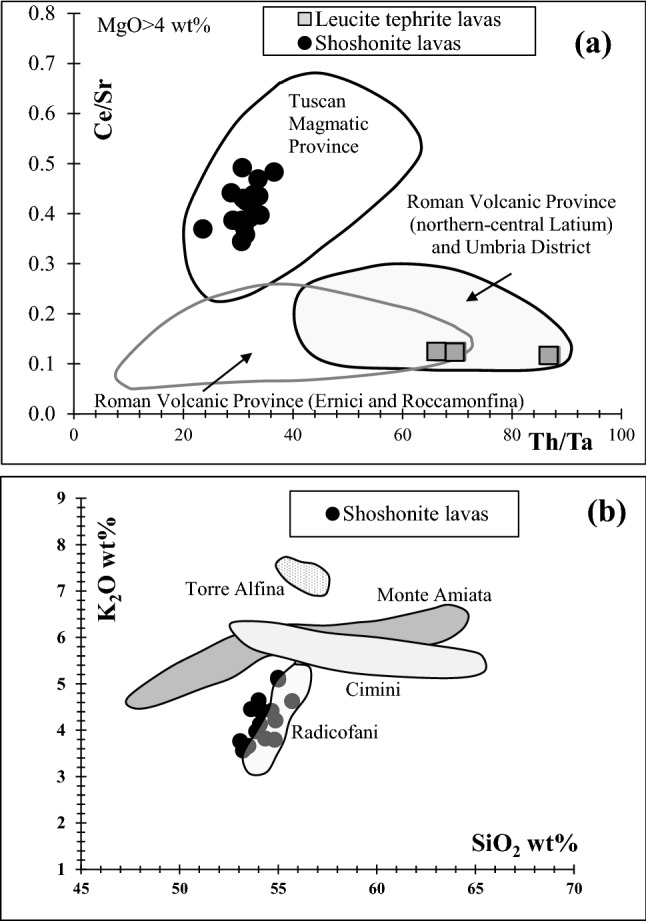
Discriminant diagrams Ce/Sr vs Th/Ta^79^ (**a**) and K_2_O vs SiO_2_^73^ (**b**).

Chemical and petrographic results clearly indicate for all the 23 samples of Monte Croce Guardia a volcanic arc (subduction-related) fingerprint and thus a provenance from the central Italy, which is also reasonable on geographic point of view and the human paths framework during the Final Bronze Age, as furtherly discussed. The TAS diagram for the products of the Tuscan Magmatic Province (Fig. 4b) clearly emphasizes a good matching of the Radicofani lavas with the most abundant investigated grinding stones of Arcevia. By contrast, the wide areal distribution of the extrusive products of the main four volcanic districts of the Roman Volcanic Province (central-northern Latium: Colli Albani, Sabatini, Vico, Vulsini; Fig. 4c) does not allow a precise attribution. If using more discriminating trace element ratios diagrams, such as the Ce/Sr vs Th/Ta plot (Fig. 6a) the leucite tephrite lavas of the grinding tools clearly fall in the field of volcanoes of northern-central Latium of the Roman Volcanic Province and Umbria District. This latter can be however ruled out on the basis of modal mineralogy and several chemical parameters which are not compatible with the investigated grinding tools^72^. Although a provenance from the Roman Volcanic Province is well defined for the leucite tephrites grinding tools, there are not peculiar mineralogic, petrographic and chemical parameters to locate, precisely their quarrying sites. Instead, the leucite phonolite (sample AR1091) shows several mineralogical, petrographic and chemical clues suggesting, very likely, a provenance from the ancient quarries of the lava lithotype near Orvieto, one of the most famous exploited sites for grinding devices at least during the entire first millennium BCE^17,37,39,58^. Besides the strong similarity about modal mineralogy and petrographic texture between sample AR1091 and the lava litothype near Orvieto (between Sugano and Buonviaggio), also some diagnostic trace elements (e.g., Th, Ba, Sr; see for comparison^17,36,38,80^) are very similar. Some other trace elements (e.g., La and Ce) are higher than the literature data of the leucite phonolite quarry near Orvieto but this can be explained by the fact the sampled analysed volume of AR1091 could have not been very representative about the proportion between leucite phenocrysts and groundmass (Fig. S2h), being the sample very friable. This could have resulted to a higher proportion of groundmass with respect leucite phenocrysts (than original ratio) in the acid digestion for chemical analysis. LREE (having very low partition coefficient for leucite^66^) could have been enriched because not diluted by proper leucite phenocryst abundance in the analysed bulk rock. The relatively low alkalis of AR1091 (9.90 wt%; Table S2) compared with standard alkalis abundance for a phonolite (generally > 11.5 wt% according to the TAS) may also account for slightly different trace element abundance of the Arcevia leucite phonolite with respect that of the quarry near Orvieto. At Frattesina di Fratta Polesine (Rovigo) archaeological settlement (Final Bronze to Iron Age; Veneto Region) some leucite phonolite grinding tools, attributed to the quarry near Orvieto (according to modal mineralogy and major elements alone) were also found^57^.

The most abundant lithotype of volcanic grinding tools found at Arcevia, namely the 17 shoshonite lava samples, are strictly compatible, on the basis of chemistry, with volcanic rocks of the Tuscan Magmatic Province, as emphasized not only in the TAS diagrams (Fig. 4a,b) but also in the Ce/Sr vs Th/Ta diagram (Fig. 6a). In addition, the K_2_O vs SiO_2_ diagram (Fig. 6b) shows a good superposition of the 17 shoshonite samples of Arcevia with the Radicofani volcanic center (southern Tuscany), ruling out the other main areas of the Tuscan Magmatic Province (Cimini, Mount Amiata, Torre Alfina). Box plots of some major (CaO, MgO) and trace (Sc, Cr, Co, Ni, Rb, Sr, Zr, Nb, Ba, La, Ce, Hf, Th) elements of the shoshonite grinding tools, compared with Radicofani rocks also confirm this provenance (Fig. S3). Modal mineralogy and petrographic texture of the 17 shoshonite grinding tools (Fig. S2a–d) closely match with those of the Radicofani shoshonites^72,73^ and thus definitively support this source area of the Tuscan Magmatic Province. Radicofani is a volcanic center consisting of a well preserved 90 m-high neck and associated lava flows^72,81^ whose compositional range is, of course, wider with respect that of the grinding tools. As a matter of fact volcanic debris of scattered isolated lava blocks, of decimetre to decametre in size, is found all around the Radicofani neck for several kilometres^73^ and it can be plausible that people from the Final Bronze Age most likely took ready-made volcanic blocks from the ground and worked or transported them to the processing sites.

### Archaeological and human movement constraints

As the most abundant investigated grinding tools come from Radicofani lavas, archaeological insights are consequential and due from this volcanic source area of Tuscany where a Final Bronze Age site coeval to that of Monte Croce Guardia is also present. The Radicofani hill is about 115 km (air-line distance) from Monte Croce Guardia. It is a highly isolated hill representing a volcanic neck^81^, which reaches ca. 890 m above sea level. The summit, a large plateau extending more than 2 hectares, ensures an exceptionally broad territorial control on the surrounding landscape. Indeed, in the eastern area of the plateau, below the Medici fortress of Medieval Age, the remains of a structure excavated in the volcanic neck, consisting of a series of post holes, have been brought to light, suggesting the existence of an oval Final Bronze Age hut, with maximum dimensions of 6 × 4 meters^82,83^. Interestingly, both villages of Monte Croce Guardia and Radicofani belong to the so-called Cetona-Chiusi-Pianello archaeological facies^1^, sharing the same pottery style and, to a certain extent, metalwork^84–86^. As it is known, this particular archaeological facies is widespread, during the Final Bronze Age, from south-west Tuscany up to Romagna^87^ and central Marche regions to the north and east, where the necropolis of Pianello di Genga and the nearby settlement of Monte Croce Guardia are located. Pottery production is characterized by the diffusion of similar shapes and decorations, the latter often presenting peculiar, engraved motifs displaying the stylized representation of two opposing waterbird heads. This decorative pattern is very common in coeval bronze work across Italy, but it is attested on pottery mainly in these areas of central Italy. It is worth to note that also bronze works show distinctive local features, especially in the field of personal adornments. One of the most important indicators of that is the so-called Casa Carletti pin, widespread from inner Tuscany up to central Marche and Romagna regions^1^.

**Figure 7:**
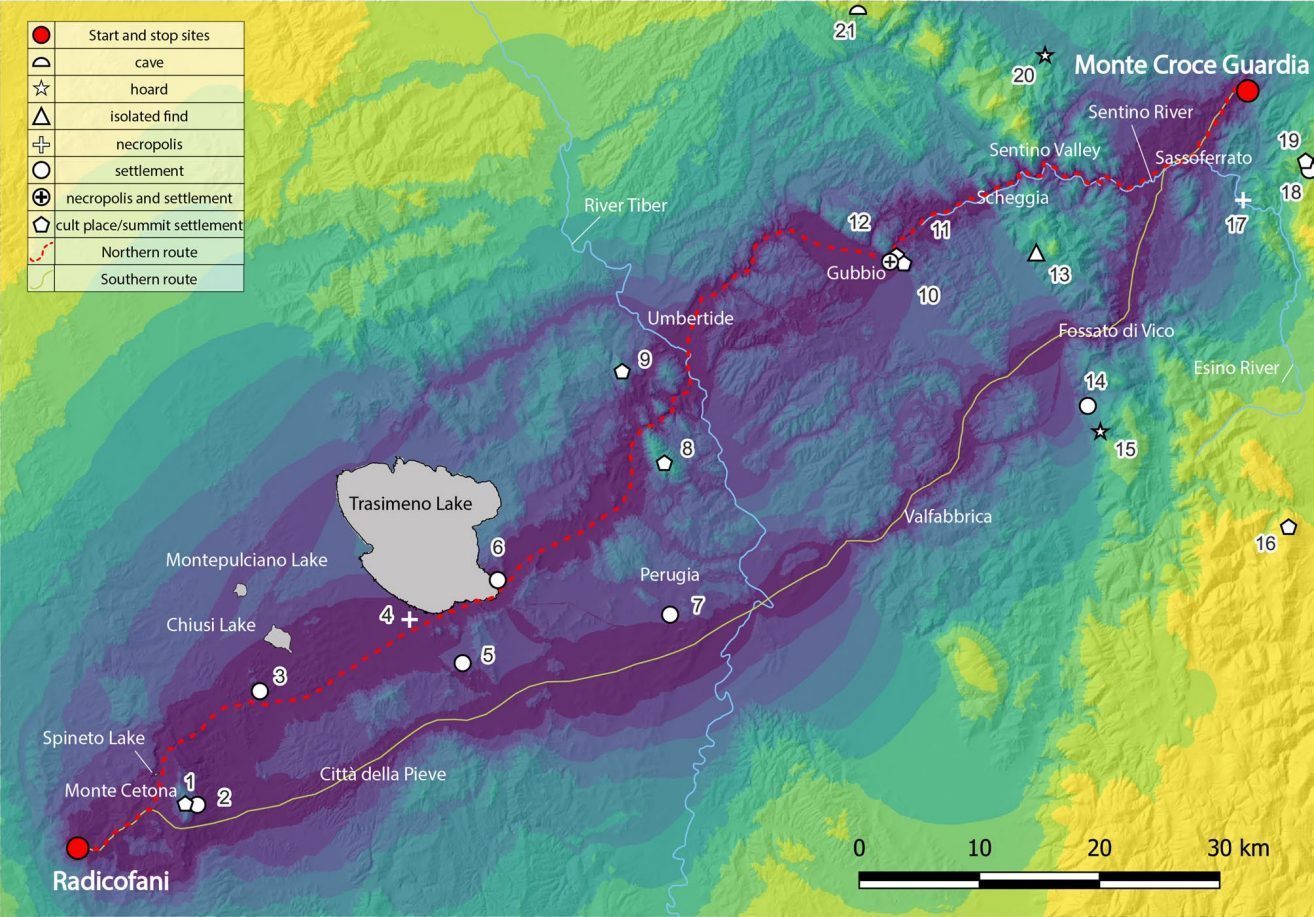
Map showing the results of the corridor analysis based on the *wcs* function. Cooler colours indicate the most convenient paths. The dashed red line (Northern route) suggests the best pathway. Numbers refer to the different contexts of the Final Bronze Age archaeological sites: 1 Monte Cetona (summit); 2 Case Carletti; 3 Chiusi; 4 Panicarola; 5 Monte Solare; 6 San Savino; 7 Perugia—Via Settevalli; 8 Monte Tezio; 9 Monte Acuto; 10 Gubbio—Via dei Consoli e Vescovado; 11 Monte Ansciano; 12 Monte Ingino; 13 Costacciaro; 14 Colle Mori; 15 Gualdo Tadino—hoard; 16 Monte Primo; 17 Pianello di Genga; 18 Gola della Rossa; 19 Monte Murano; 20 Frontone—Chiuse; 21 Fondarca—Grotta delle Nottole.

The sharing of specific cultural traits suggests a high degree of connectivity among the communities were living in the regions mentioned above. Nevertheless, the social and economic processes and interrelations among these communities are not yet fully understood. In the case of pottery, we can imagine an intense circulation of models; the same could happen also for metalwork, in this case along with the possible mobility of specialised artisans spreading models and possibly raw materials. As mentioned, craft productions testify a strong degree of shared cultural models and circulation of raw materials. To deeply explore this phenomenon, an analysis of the geographical distribution of the known coeval sites of the area between Radicofani and Monte Croce Guardia, compared with the possible routes linking both sides of the Apennine Mountains was performed through the DEM TINITALY/01^88^. The various tiles involved in the analysis (W47570; W47070; W47575; W47075; W47580; W47080; W48070; W48075; W48080) are first merged and, subsequently a rectangular frame, including the location of the archaeological sites of Radicofani at one edge and Monte Croce Guardia at the other, have been extracted. The DEM, originally at 10 m resolution, is then resampled into a new raster at 50 m resolution. The aim of this down sampling is to reduce the number of cells and consequently the amount of data to be processed. The cells of the raster corresponding to the Lake Trasimeno basin, the largest body of water in the analysed area, have been eliminated using the current shoreline limits as a reference (using geographical information from the OpenStreetMap project—https://www.openstreetmap.org/). No other friction factors have been used to investigate patterns of human movement; in our analysis the difficulty of movement is determined only by the terrain slope. Analyses have been carried out using Alberti’s Movecost package^8^. This package, developed for the scripting and statistical analysis software R (https://www.r-project.org/), includes different human-dependent cost-functions which can be applied to determine non-isotropic accumulated cost surface, least-cost paths, least-cost corridors and least-cost networks. Cost-functions are divided into different types according to the method of cost determination including those expressing cost in travel time, metabolic energy expenditure, and dimensionless cost^89^. The functions expressing cost in terms of walking time have been mainly used to estimate the time-distance between Radicofani and Monte Croce Guardia through the elaboration of an anisotropic accumulated cost from the starting location (Radicofani) to the final destination (Monte Croce Guardia). The wheeled-vehicle critical slope cost function (*wcs*), which simulates the movement using a wagon^10,89^ have been instead used, by performing a corridor analysis (movecorr^89^), to determine the most convenient routes between starting and arrival points. The results of the corridor analysis using the *wcs* are provided in discrete form (specifically through the transformation of the continuous distribution of values into 10 quantiles) in the map of Fig. 7 where cooler colours represent the lower cost surfaces, while warmer colours represent the higher cost surfaces. The Northern route represents the best path between the two contexts (dashed red line in Fig. 7) passes north of Monte Cetona, skirts Trasimeno Lake and ascends north to the present-day cities of Umbertide, Gubbio, Scheggia and following the course of the Sentino valley to Sassoferrato. It is worth to note this Northern route could also take advantage by several water supplies such as Spineto and Chiusi Lakes and Tiber and Sentino Rivers (Fig. 7) besides, of course, Trasimeno Lake. The Southern route skirts Monte Cetona to the south and passes the present-day cities of Città della Pieve, Perugia, Valfabbrica and Fossato di Vico. The route then ascends to the north where it would meet the Northern route at Sassoferrato. The Northern route is approximately 140 km long, while the Southern route is 128 km long. Despite its length, the algorithm considers the Northern route to be more suitable for a wagon route. A spatial correlation between the Northern route and the Final Bronze Age known sites of the area seems evident. The link between the sites and the routes is simply quantifiable by measuring the linear distance between the sites and the nearest route. The results are summarised by the boxplot of Fig. S4. The median distance between all the considered Final Bronze Age sites and the Northern route is 2 km, while it increases to 5 km in the case of the Southern route. Furthermore, most of the known Final Bronze Age sites are less than 5 km distant from the Northern route (Fig. 7).

As stated, the *wcs* algorithm returns a dimensionless value to quantify the cost, whereas it would be useful to have an estimate of the travel time in hours. In this way 11 algorithms of the Movecost package^8^ have been used to calculate the walking time between Radicofani and Monte Croce Guardia (Fig. S5 and Table S3). Most of the algorithms identify the shortest path along an almost straight line between the two sites. Marquez-Perez function, Uriarte Gonzalez’s function and Marin Arroyo’s function identify the shortest paths that fit with the Southern route; only the Rees hiking function indicates a shortest path almost congruent with the Northern route. A boxplot summarizes the results (Fig. S6) and clearly shows how most of the algorithms give a result of approximately 25–30 h which are only an approximation of the needed travel (walking) time. Indeed, this evaluation (Table S3) is subject to several factors that cannot always be easily assessed. Especially in the case of heavy load transports, velocity depends mainly on the following issues: (1) the vector, humans, animals, or wheel chariots; (2) the existence of roads; (3) the total weight of the carried items. It is worth to note that the most probable “best-path” (Northern route), identified through the *wcs* algorithms, takes into account the morphology (slope) of the terrain as the only parameter. This algorithm considers a critical slope above which hairpin turns are more effective than direct ascent or descent that well fit with a chariot or the walking of a loaded animal. As a matter of fact, pack animals are attested in the Late Bronze Age, and bone remains probably pertaining donkeys or mules have been found also at Monte Croce Guardia. In addition, the easiness of movement (and thus speed) along the proposed routes depends also on the ground conditions, spanning from hardly walkable areas such as woodlands or wetlands, to proper roads. During the Middle/Late Bronze Age the existence of roads connecting the sites is very likely, as suggested, e.g., by the pathway found in northern Italy (near Verona)^90^ but in general clues are very difficult to find because of very bad conservation of the related archaeological traces.

It can be thus inferred that the Northern route was probably travelled using pack animals (and wheel chariots) with an estimated time constrained to the average human speed.

## Conclusions

This comprehensive igneous petrology study clearly indicates that the most abundant volcanic fragments of grinding tools from the Final Bronze Age settlement of Monte Croce Guardia come from the shoshonite (K-series) lavas of Radicofani. This area of provenance and the context of the final destination of the grinding tools appear to be connected by two routes (the northern and southern ones, nearly parallel each other) whose paths seem to be correlated with the presence of numerous coeval sites, suggesting the existence of a well-organized territory, arranged on a series of strongly interconnected communities living on both sides of the central Apennine Mountains. The approach using algorithms consider the Northern route (approximately 140 km long) as the best fitting pathway probably travelled by pack animals (and wheel chariots) in an estimated travel time of 25–30 h walking. The presence of several water supplies along this route (lakes and rivers) may have also contributed to choose the pathway to go. The Apennine Mountains did not therefore represent a barrier limiting movements for people of three thousand years ago. The Final Bronze Age cultural matrix of the investigated corridor area of central Italy seems to have been corresponding to a shared pattern of sites distribution that privileged well defended locations along the most convenient routes. The presence at Monte Croce Guardia of several fragments of volcanic grinding tools whose petrological constraints clearly demonstrate a provenance from the Radicofani area, sheds light on some other possible patterns of interactions between these far away communities of central Italy. These communities could be probably also focused on the exchange of raw materials and final products aimed at the best performance of strategic economic activities, such as the transformation of cereals and the production of flours. Seeking this line of reasoning we can speculate different types of relationships among local communities. Thinking about the possibility that grain milling activities were carried out at the household level possibly by women, we cannot rule out that the circulation of millstones made by allochthon raw materials were also linked to some forms of marriage exchange. Of course, this hypothesis would have to be supported by data from further types of analyses, such as isotope analyses carried out on the human bones from funeral coeval sites. Regarding the provenance areas of the remaining, less abundant volcanic grinding tools of the High-K series (leucite tephrites and phonolite), at the current state of knowledge it is not possible to pinpoint the same level of interaction with Monte Croce Guardia site. Their provenance from the Roman Magmatic Province (Latium) is however well proved through mineralogical and chemical constraints and, at least for the leucite phonolite sample, a provenance from the well-known quarrying site near Orvieto (between the localities of Buonviaggio and Sugano) is very likely.

## Supplementary Information

Supplementary Information 1.

Supplementary Information 2.

## Data Availability

All data generated or analysed during this study are included in this published article (and its supplementary information files).

## References

[1] Cardarelli, A. *et al.* Nuove ricerche nell’abitato della tarda età del Bronzo di Monte Croce Guardia (Arcevia - AN): scavi 2015–2016. *Rivista di Scienze Preistoriche*, **LXVII**, 321–380 (2017).

[2] Cardarelli, A., Bettelli, M. & Di Renzoni, A. Prima dei Piceni. Lo scavo dell'abitato di Monte Croce Guardia nel quadro delle dinamiche storiche comprese fra la fine del II e l'inizio del I millennio a.C. in Italia centrale. *Archeologia Picena.* Frapiccini N. & Naso A. (eds.) Atti del convegno internazionale di studi, Ancona 14–16.11.2019, Roma 2022, 13–33 (2022). ISBN: 978-88-5491-321-9.

[3] Conticelli S (2009). Shoshonite and sub-alkaline magmas from an Ultrapotassic Volcano: Sr-Nd-Pb isotope data on the Roccamonfina volcanic rocks, Roman Magmatic province, southern Italy. Contrib. Mineral. Petrol..

[4] Bell T, Wilson A, Wickham A (2002). Tracking the Samnites: Landscape and communications routes in the Sangro valley Italy. Am. J. Archaeol..

[5] Whitley TG, Hicks LM (2003). A Geographic Information Systems Approach to Understanding Potential Prehistoric and Historic Travel Corridors. Southeastern Archaeol..

[6] Silva F, Steele J (2012). Modeling boundaries between converging fronts in prehistory. Advs. Complex Syst..

[7] Bicho, N., Cascalheira, J. & Gonçalves, C. Early Upper Paleolithic colonization across Europe: Time and mode of the Gravettian diffusion. *PLoS ONE* **12**(5), e0178506. 10.1371/journal.pone.0178506 (2017). 10.1371/journal.pone.0178506 PMC5443572 28542642

[8] Alberti, G. Movecost: An R package for calculating accumulated slope-dependent anisotropic cost-surfaces and least-cost paths. *SoftwareX,* **10** (2019).

[9] Surface-Evans, S.L. & White, D.A. Least Cost Analysis of Social Landscapes: Archaeological Case Studies. *University of Utah Press*, (2012).

[10] Herzog, I. The Potential and Limits of Optimal Path Analysis, in Bevan A. & Lake M. (eds.), *Computational Approaches to Archaeological Spaces.* Routledge, (2013).

[11] Herzog I (2014). A review of case studies in archaeological least-cost analysis. Archeologia e Calcolatori.

[12] Herzog I (2022). Issues in replication and stability of least-cost path calculations. SDH.

[13] Moritz, L.A. *Grain-mills and flour in classical antiquity*. 230 p. (Oxford at the Clarendon Press, 1958).

[14] Peacock DPS (1980). The Roman millstone trade: a petrological sketch. World Arch..

[15] Williams-Thorpe O (1988). Provenancing and archaeology of Roman millstones from the Mediterranean Area. J. Arch. Sci..

[16] Curtis, R.I. *Ancient food technology*. (eds. Lucas, A. and Walton, S.A.) 477 p. (Boston Brill, 2001).

[17] Santi, P., Antonelli, F., Renzulli, A. & Pensabene, P. Leucite phonolite millstones from the Orvieto production centre: new data and insights into the Roman trade. *Per. Min.**Spec. Issue 3 Showcase Ital. Res. Appl. Petrol.* **73**, 57**–**69 (2004).

[18] Hockensmith, C.D. *The millstone industry: a summary of research on quarries and producers in the United States, Europe and elsewhere*. (McFarland & Company, Inc., Publishers), 269 p (Jefferson, North Carolina and London, 2009).

[19] Williams, D. & Peacock D. (eds.) *Bread for the People: the archaeology of mills and milling*, *Archaeopress*, 380 p. (University of Southampton, Series in Archaeology, 2011).

[20] Alonso, N. & Frankel, R. A Survey of Ancient Grain Milling Systems in the Mediterranean, in *Les meules du Néolithique à l’époque médiévale: Technique, culture, diffusion* (Buchsenschutz, O., Lepareux-Couturier, S. & Fronteau, G. eds.) 461–478 (2017).

[21] Bloxam, E. Visualizing the invisible: re-discovering the ancient grinding stone quarries of the Aswan West Bank, Egypt, in *Bread for People: The Archaeology of Mills and Milling,* Williams, D., Peacock, D. (eds.). Proceedings of a Colloquium Held in the British School at Rome, 4–7 November 2009. Series in Archaeology No.3. Bar International Series 2274 University of Southampton, 43–53 (2011).

[22] Elliott, C., Xenophontos C. & Malpas J.G. Petrographic and mineral analyses used in tracing the provenance of Late Bronze Age and Roman artefacts from Cyprus. *Rep. Deptm. Antiqu. Cyprus*, 80–96 (1986).

[23] Renzulli, A., Santi, P., Gambin T. & Bueno Serrano P. Pantelleria Island as a center of production for the Archaic Phoenician trade in basaltic millstones: new evidence recovered and sampled from a shipwreck off Gozo (Malta) and a terrestrial site at Cadiz (Spain). *J. Arch. Sci. Rep.* **24**, 338–349 (2019).

[24] Piperno, D.R., Weiss, E., Holst, I. & Nadel, D. Processing of wild cereal grains in the Upper Palaeolithic revealed by starch grain analysis. *Nature*, **430** (2004). 10.1038/nature02734 15295598

[25] White D (1963). A survey of millstones from Morgantina. Am. J. Arch..

[26] Williams-Thorpe O, Thorpe RS (1993). Geochemistry and trade of eastern Mediterranean millstones from the Neolithic to Roman Periods. J. Arch. Sci..

[27] Frankel R (2003). The Olynthus mill, its origin and diffusion: Typology and distribution. Am. J. Arch..

[28] Childe VG (1943). Rotary querns on the continent and in the Mediterranean basin. Antiquity.

[29] Py, M. Meules d’époques protohistorique et romaines provenant de Lattes, in *Lattara 5. Recherches sur l’économie vivrière des Lattarenses* (Py, M. ed.) 184–230 (1992).

[30] Williams-Thorpe O, Thorpe RS (1990). Millstone provenancing used in tracing the route of a fourth-century BC Greek merchant ship. Archaeometry.

[31] Santi P, Renzulli A, Bell M (2015). The volcanic millstones from the archaeological site of Morgantina (Sicily): Provenance and evolution of the milling techniques in the Mediterranean area. Archaeometry.

[32] Mayesche, B. A Pompeian bakery on the via dell’Abbondanza, in *Studia Pompeiana and Classica in honour of Wilhelmina Jashemski*, R.I. Curtis (ed.), 1 A.D. Caratzas, New Rochelle, NY,149–166 (1988).

[33] Peacock DSP (1989). The mills of Pompeii. Antiquity.

[34] McCallum, M. The supply of stone to the city of Rome: A case study of the transport of anician building stone and millstone from the Santa Trinità quarry (Orvieto), in *Trade and Exchange*, C.D. Dillan, C.L. White (eds.) Springer Berlin, **5** (2010).

[35] Wefers, S. Still using your saddle quern? A compilation of the oldest known rotary querns in Western Europe, in *Bread for the People: the archaeology of mills and milling*, D. Williams, D. Peacock (eds.), Archaeopress, University of Southampton, Series in Archaeology, **3**, 67–76 (2011).

[36] Santi, P. Gambin T. & Renzulli, A. The millstone trade from the most exploited Italian volcanic areas: an overview from the Phoenicians to the roman period. *Ann. Geoph*. **64**(5), VO551. 10.4401/ag-8647 (2021).

[37] Peacock DPS (1986). The production of Roman millstones near Orvieto, Umbria Italy. Antiquity. J..

[38] Antonelli F, Nappi G, Lazzarini L (2001). Roman millstones from Orvieto (Italy): Petrographic and geochemical data for a new archaeometric contribution. Archaeometry.

[39] Renzulli A, Santi P, Nappi G, Luni M, Vitali D (2002). Provenance and trade of volcanic rock millstones from Etruscan-Celtic and Roman archaeological sites in Central Italy. Eur. J. Min..

[40] Buffone L, Lorenzoni S, Pallara M, Zanettin EL (1999). macine rotatorie in rocce vulcaniche di Pompei. Riv. St. Pompeiani.

[41] Buffone L, Lorenzoni S, Pallara M, Zanettin E (2003). The millstones of Ancient Pompei: A petro-archaeometric study. Eur. J. Miner..

[42] Lorenzoni S, Pallara M, Vanturo D, Zanettin E (1996). Archaeometric preliminary study of volcanic millstones from Neolithic-Roman archaeological sites of the Altamura area (Apulia, Southern Italy). Sci. Technol. Cult. Herit..

[43] Lorenzoni S, Pallara M, Zanettin E (2000). Volcanic rock Bronze Age millstones of Apulia, Southern Italy: Lithology and Provenance. Eur. J. Min..

[44] Santi P, Chaigneau C, Renzulli A (2022). Petrological footprints of the millstones of Megara Hyblaea (Sicily Island, Italy) highlight the human interactions with Mediterranean volcanoes. Sci. Rep..

[45] Williams-Thorpe O, Thorpe RS (1991). Millstones that mapped the Mediterranean. New Sci..

[46] Santi P, Renzulli A, Gullo R (2013). Archaeometric study of the hopper-rubber and rotary Morgantina-type volcanic millstones of the Greek and Roman periods found in the Aeolian archipelago (southern Italy). Eu. J. Min..

[47] Schwall, C. & Gluhak, T.M. The volcanic rock grinding stones from Selinunte, Sicily (Italy): Archaeological evidence and geochemical provenance analyses, in *Tilting at mills: The Archeology and Geology of Mills and Milling,* (Alonso, N. & Anderson, T.J. eds.) *Revista d’arqueologia de Ponent extr. 4,* 213–222 (2019).

[48] Antonelli, F., Columbu, S., de Vos Raaijmakers, M. & Andreoli, M. An archaeometric contribution to the study of ancient millstones from the Mulargia area (Sardinia, Italy) through new analytical data on volcanic raw material and archaeological items from Hellenistic and Roman North Africa. *J. Arch. Sci.* **50**, 243–261 (2014).

[49] Peacock, D.P.S. Archaeology of Pantelleria. *Nat. Geogr. Soc.* 1977 projects. Res. Rep. 567–579 (1985).

[50] Di Bella M (2018). Archeometric characterization of prehistoric grindstones from Milazzo Bronze Age settlement (Sicily, Italy). Archaeol. Anthropol. Sci..

[51] Santi, P., Foresta Martin, F., Spatafora, F., De Vita, S. & Renzulli, A. Volcanic Grinding Tools in Ustica Island (Tyrrhenian Sea, Italy): Local Production vs. Import of Morgantina-Type Millstones in the Hellenistic-Roman Period. *Minerals* **10**, 389 (2020). 10.3390/min10050389

[52] Xenophontos C, Elliot C, Malpas JG (1988). Major and trace-element geochemistry used in tracing the provenance of late Bronze Age and Roman basalts artefacts from Cyprus. Levant.

[53] Katerinopulos, A., Kokkorou-Alevras, G., Mavrogonatos, K. & Poupaki, E. Volcanic millstones from ancient Halasarna, Kos Island. *J. Hell. Rom. Mat. Cult*. **5.2.2**, 161–196 (2016).

[54] Di Bella M (2016). Archaeometric characterization of Roman volcanic millstones from Messina territory (Sicily, Italy). Per. Min..

[55] Ferla R, Alaimo G, Falsone F, Spatafora F (1984). Studio petrografico delle macine di età arcaica e classica da Monte Castellazzo di Poggioreale. Sicilia Archaeol..

[56] Peacock, D.S.P. Iron Age and Roman quern production at Lodsworth, west Sussex. *Antiquar. J.* **LCVII**, Part I. (1987).

[57] Cattani M, Lazzarini L, Falcone R (1997). Macine protostoriche dall’Emilia e dal Veneto: Note archeologiche, caratterizzazione chimico-petrografica e determinazioni della provenienza. Padusa.

[58] Antonelli, F., Nappi, G. & Lazzarini L. Sulla “pietra da mole” della regione di Orvieto. Caratterizzazione petrografica e studio archeometrico di macine storiche e protostoriche dall’Italia centrale, in *Proceedings of “I° Congresso Nazionale di Archeometria”*, Verona, 1999, 195–207, Patron ed. (2000).

[59] Antonelli, F., Bernardini, F., Capedri, S., Lazzarini, L. & Montagnari Kokelj E. Archaeometric study of protohistoric grinding tools of volcanic rocks found in the Karst (Italy-Slovenia) and Istria (Croatia). *Archaeom*etry, **46**(4), 537–552 (2004).

[60] Le Bas MJ, Le Maitre RW, Streckeisen A, Zanettin B (1986). A chemical classification of volcanic rocks based on the total alkali-silica diagram. J. Petrol..

[61] Alagna, K.E, Peccerillo, A., Martin, S., Donati, C. Tertiary To Present Evolution Of Orogenic Magmatism In Italy. *J. of the Virtual Explorer*, **36**, paper 18 (2010).

[62] Le Maitre, R.W. *et al. Igneous rocks. A classification and glossary of terms*. (ed. Le Maitre, R.W.) 193 p. (Blackwell Scientific Publications, 1989).

[63] Streckeisen, A. Classification and nomenclature of igneous rocks. Final report of an inquiry. *Neus Jahrb. Mineral. Abh.*, **107**, 144–240 (1967).

[64] Sun, S.S. & McDonough, W.F. Chemical and isotopic systematics of oceanic basalts: implications for mantle composition and processes, in *Magmatism in Ocean Basins* (eds. Saunders, A.D., Norry, M.J.) *Geol. Soc. London*, *Sp. Publ*. **42**, 313**–**345 (1989).

[65] Winter, J.D. *Principles of Igneous and Metamorphic Petrology*, 2nd Ed. (Whitman College 2010).

[66] Rollinson*,* H.R*.* Using Geochemical Data*: Evaluation, Presentation, Interpretation. 352 p. (Longman* Geochemistry Series, 2021).

[67] Wood DA, Joron JL, Treuil M (1979). A reappraisal of the use of trace éléments to classify and discriminate between magma series erupted in different tectonic settings. Earth Planet. Sci. Letters.

[68] Peccerillo, A. *Plio-quaternary volcanism in Italy*. 365 p. (Springer Verlag, 2005).

[69] Lustrino M, Wilson M (2007). The circum-Mediterranean anorogenic Cenozoic igneous province. Earth Sci. Rev..

[70] Francalanci, L., Vougioukalakis, G. E., Perini, G. & Manetti, P. A West-East traverse along the magmatism of the south Aegean volcanic arc in the light of volcanological, chemical and isotope data, in *The South Aegean Active Volcanic Arc* (eds Fytikas, M. & Vougioukalakis, G. E.) 65–111 (Elsevier, 2005).

[71] Avanzinelli, R., Lustrino, M., Mattei, M., Melluso, L. & Conticelli S. Potassic and ultrapotassic magmatism in the circum-Tyrrhenian region: The role of carbonated pelitic vs. pelitic sediment recycling at destructive plate margin. *Lithos*, **113**, 213–227 (2009).

[72] Conticelli, S. et al. Leucite-bearing (kamafugitic/leucititic) and -free (lamproitic) ultrapotassic rocks and associated shoshonites from Italy: Constraints on petrogenesis and geodynamics. *J. Virtual Explorer* **36**, paper 20 (2010).

[73] Conticelli S, Avanzinelli R, Marchionni S, Tommasini S, Melluso L (2011). Sr-Nd-Pb data from the Radicofani volcano, Central Italy: Constraintson heterogeneities in a veined mantle responsible for the shift from ultrapotassic shoshonite to basaltic andesite magmas in a post-collisional setting. Miner. Petrol..

[74] Beccaluva L, Bonadiman C, Coltorti M, Salvini L, Siena F (2001). Depletion events, nature of metasomatizing agent and timing of enrichment processes in lithospheric mantle xenoliths from the VVP. J. Petrol..

[75] Beccaluva L, Bianchini G, Bonadiman C, Coltorti M (2007). Intraplate lithospheric and sublithospheric components in the Adriatic domain: Nephelinite to tholeiite magma generation in the Paleogene Veneto Volcanic Province Southern Alps. Geol. Soc. Am..

[76] Civetta L, D’Antonio M, Orsi G, Tilton GR (1998). The Geochemistry of Volcanic Rocks from Pantelleria Island, Sicily Channel: Petrogenesis and Characteristics of the Mantle Source Region. J. Petrol..

[77] Avanzinelli R, Bindi L, Menchetti S, Conticelli S (2004). Crystallisation and genesis of peralkaline magmas from Pantelleria volcano, Italy: An integrated petrological and crystal-chemical study. Lithos.

[78] White, J.C., Parker, D.F. & Minghua Ren, M. The origin of trachyte and pantellerite from Pantelleria, Italy: Insights from major element, trace element, and thermodynamic modelling. J*. Volcanol. Geoth. Res.,* **179**, 33–55 (2009).

[79] Peccerillo A (2003). Plio-Quaternary magmatism in Italy. Episodes.

[80] Antonelli F, Lazzarini L (2010). Mediterranean trade of the most widespread Roman volcanic millstones from Italy and petrochemical markers of their raw materials. J. Arch. Sci..

[81] Perugini, D. & D’Orazio, M. Radicofani. *“Miocene to Recent Plutonism and Volcanism in the Tuscan Magmatic Province (Central_Italy)”; Per. Min., Special Issue* (Poli, G., Perugini, D., Rocchi S. & Dini A. Guest_Editors), 361 p (2003).

[82] Rossi, S. Lo scavo della fortezza. La fase protostorica (XII-X secolo a.C.), in *La città fortificata di Radicofani*, Avetta C. (ed.), Siena, 149–153 (1998).

[83] Rossi, S. Capanna del Bronzo Finale a Radicofani, Siena, in *Preistoria e Protostoria della Toscana*, Atti XXXIV Riunione Scientifica IIPP, Firenze, 29 settembre-2 ottobre 1999, 579–582 (2001).

[84] Zanini, A. L’età del Bronzo finale nella Toscana interna alla luce delle più recenti acquisizioni. *Rivista di Scienze Preistoriche,* **XLVI**, 87–144 (1994).

[85] Bietti Sestieri, A.M., De Angelis, M.C., Negroni Catacchio, N., Zanini, A. La Protostoria della Toscana dall’età del Bronzo recente al passaggio alla prima età del ferro, in *Preistoria e Protostoria della Toscana*, Atti XXXIV Riunione Scientifica IIPP, Firenze, 29 settembre-2 ottobre 1999, 117–166 (2001).

[86] Peroni, R. La Toscana nel contesto peninsulare durante la Protostoria, in *Preistoria e Protostoria della Toscana*, Atti XXXIV Riunione Scientifica IIPP, Firenze, 29 settembre-2 ottobre 1999, 293–305 (2001).

[87] La Pilusa E, Zanini AL (2009). Romagna tra la fine del mondo terramaricolo e nuovi assetti medio-tirrenici: il sito di Ripa Calbana. IpoTESI di Preistoria.

[88] Tarquini, S., Isola, I., Favalli, M., Mazzarini, F., Bisson, M., Pareschi, M.T. & Boschi E. TINITALY/01: A new Triangular Irregular Network of Italy. *Ann. Geophys.* **50** (2009).

[89] Alberti, G. Movecost: Calculation of slope-dependant accumulated cost surface, least-cost paths, least-cost corridors, least-cost networks related to human movement across the landscape (2022).

[90] Gonzato, F. & Nuvolari, S. Una strada strutturata dell’età del Bronzo a Vallese di Oppeano, in *Verona e le sue strade, archeologia e valorizzazione*, Basso, P., Bruno, B., Cenci C. & Grossi P. (eds.), Cierre Edizioni, Verona, 81–88 (2019).

