# Supplementary material for: Tracing the human movements of three thousand years ago by volcanic grinding tools in the Final Bronze Age settlement of Monte Croce Guardia (Arcevia-Marche Region, central Italy)
Source: Sci Rep. 2023 Apr 29;13:7022. doi: 10.1038/s41598-023-34033-x (PMC10148880; doi:10.1038/s41598-023-34033-x)
Supplement: Supplementary file 1 — Supplementary Information 1. [file 41598_2023_34033_MOESM1_ESM.pdf]

Trenches 1 e 2

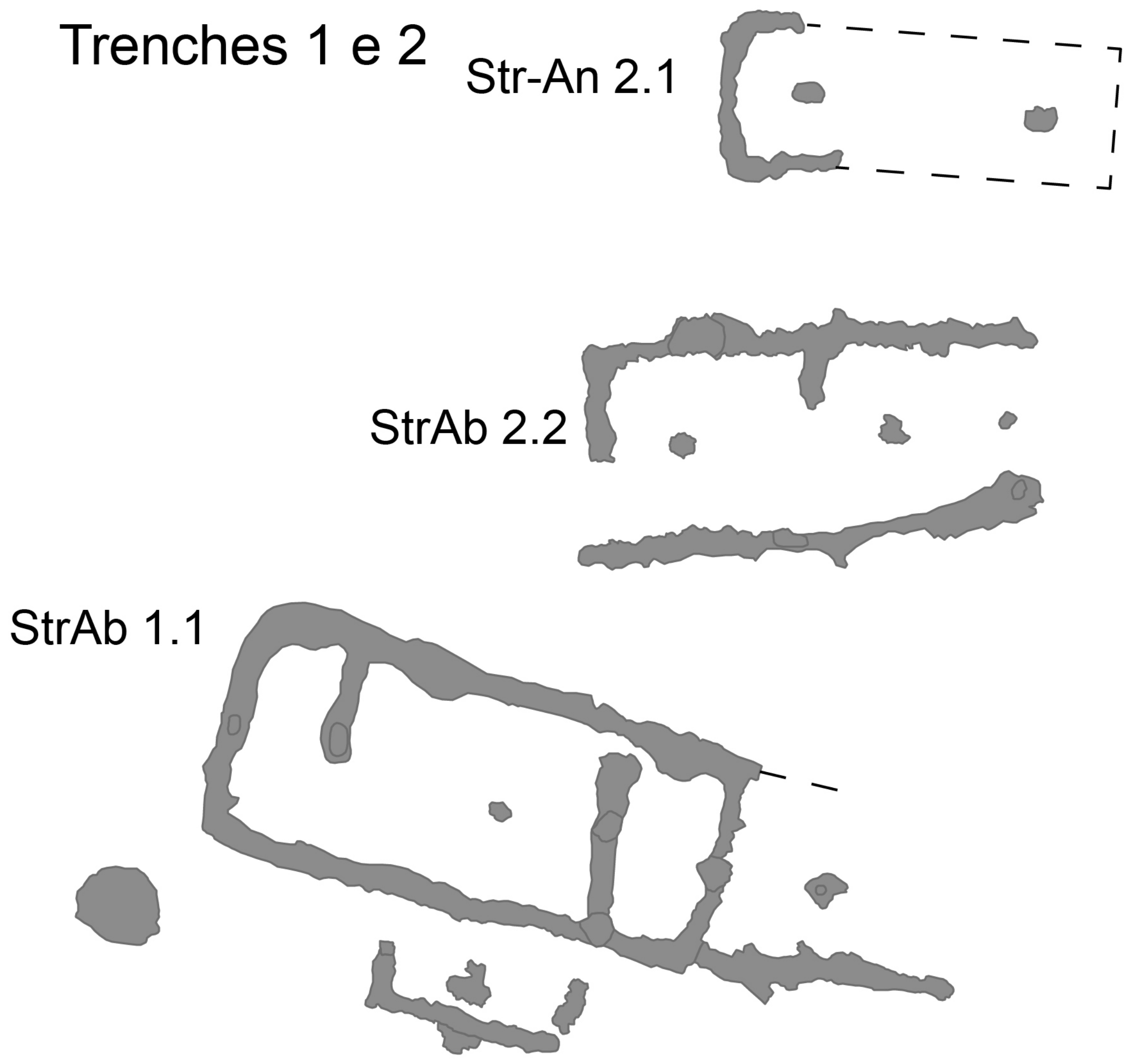

	Int.	Ext.
**Str-Ab 1.1**		
Area	77 m²	104 m²
Length	16 m	17,2 m
Width	5 m	6,8 m
**Str-An 2.1**		
Area	30 m²	40 m²
Length	10 m	10 m
Width	3,3 m	4 m
**Str-Ab 2.2**		
Area	48 m²	65 m²
Length	11 m	12 m
Width	4,3 m	5 m

Trench 4

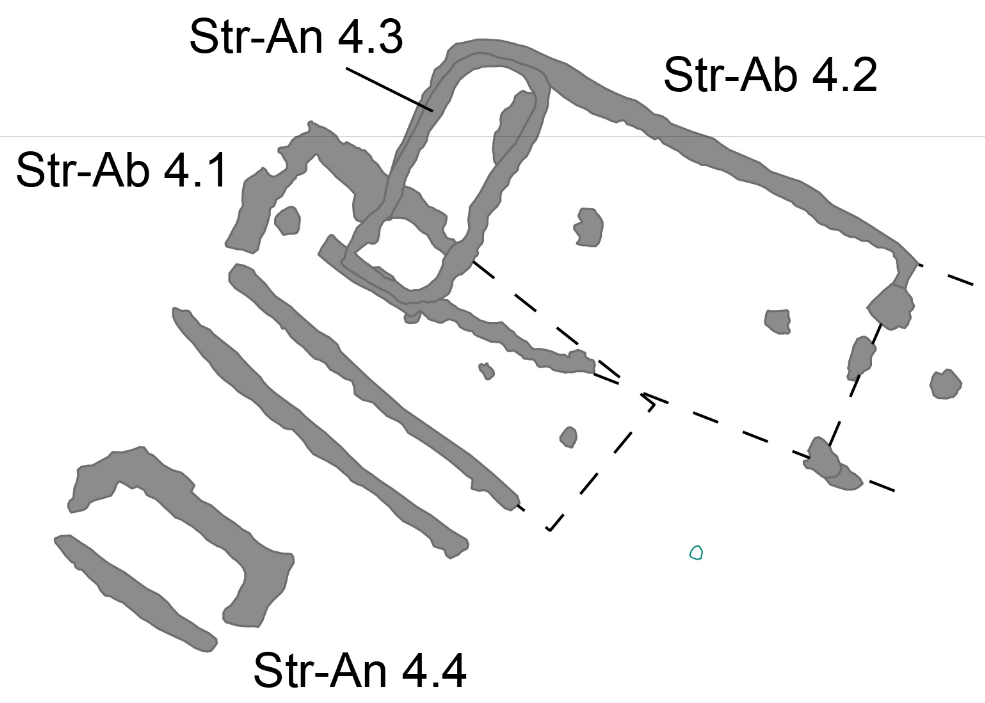

	Int.	Est.
**Str-Ab 4.1**		
Area	41 m²	52 m²
Length	10,5 m	10 m
Width	4 m	5 m
**Str-Ab 4.2**		
Area tot	90 m²	110 m²
Length	16 m	16 m
Width	6,2 m	7 m
**Str-An(?) 4.3**		
Area tot	12 m²	20 m²
Length	6,4 m	7 m
Width	2 m	3 m
**Str-An 4.4**		
Area	9 m²	19,8 m²
Length	4,5 m	6 m
Width	2 m	3,5 m

Trench 6

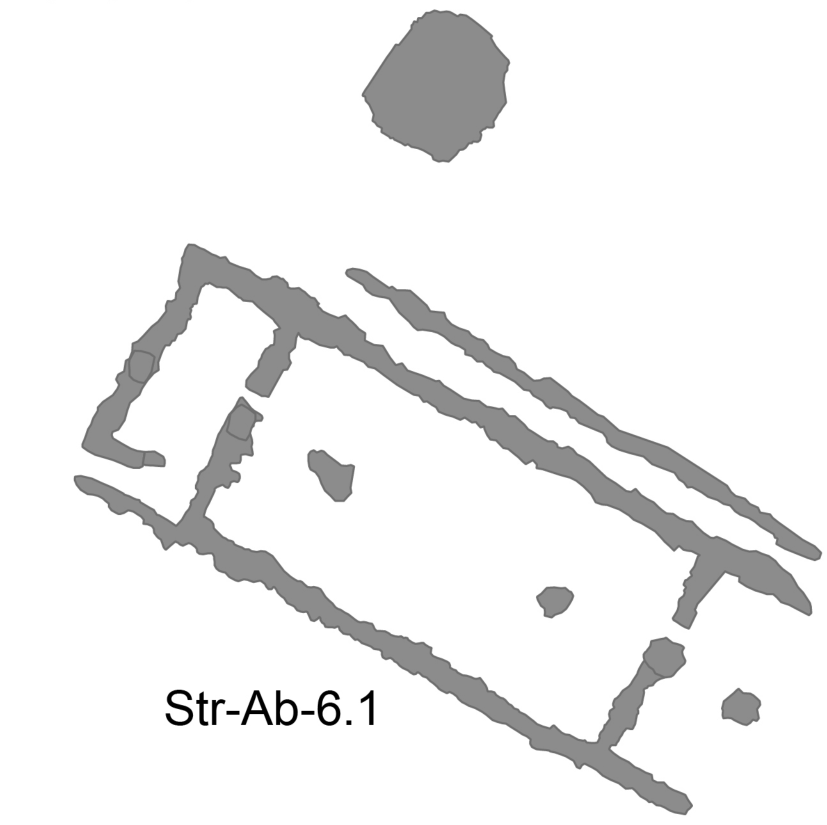

	Int.	Ext.
**Str-Ab 6.1**		
Area	101,5 m²	133 m²
Length	18 m	20 m
Width	5,7 m	7 m

Str-Ab = Abitation
Str-An = Ancillary Structure
Int. = measures relative to the interior
Ext. = measures comprising the perimetral foundations of the structures

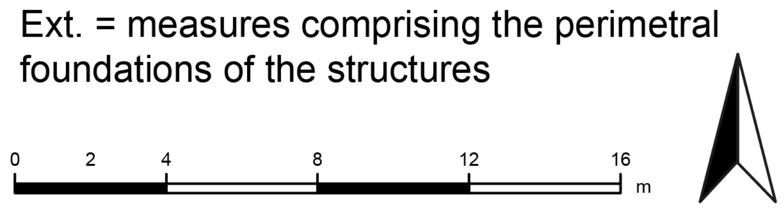

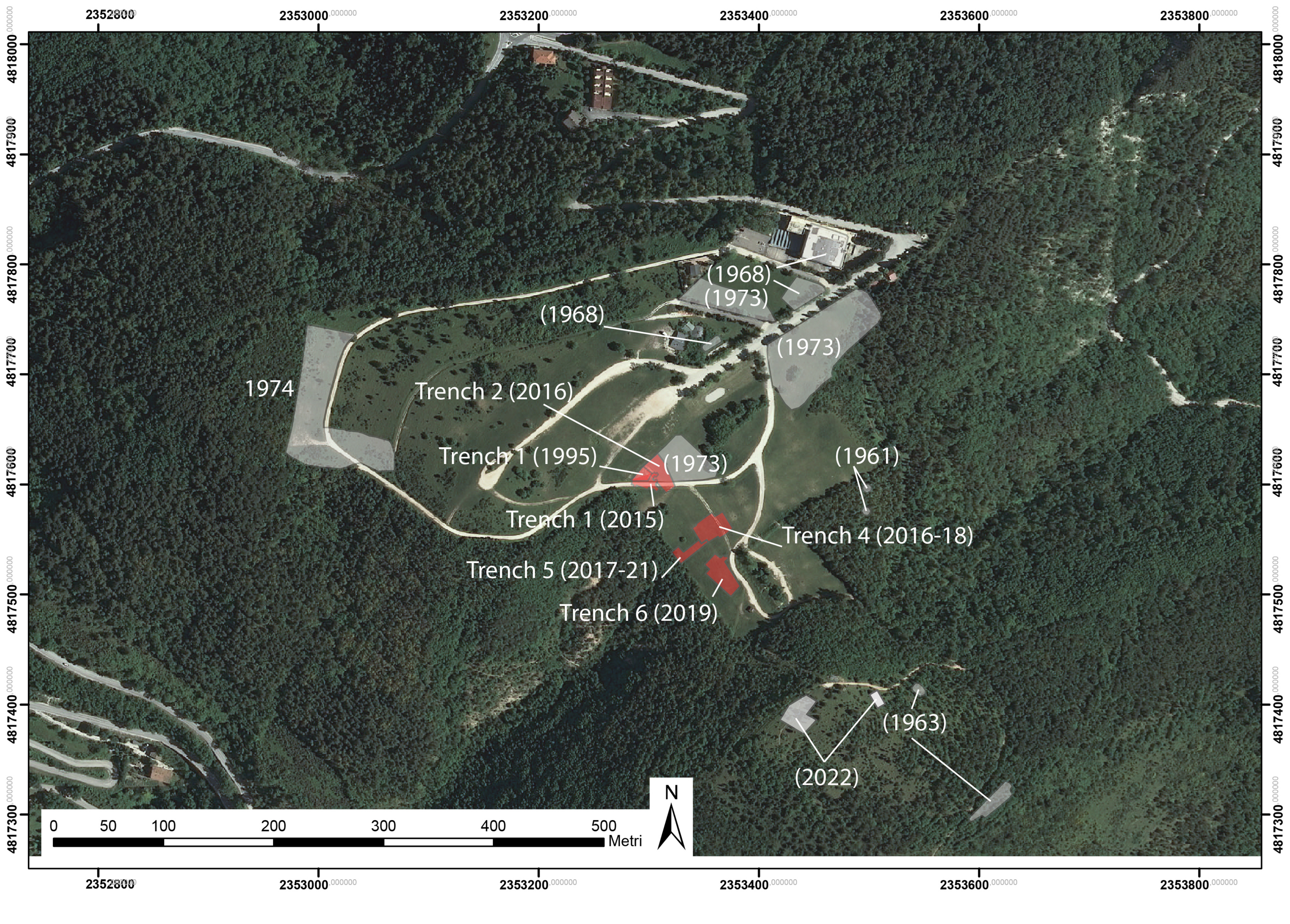

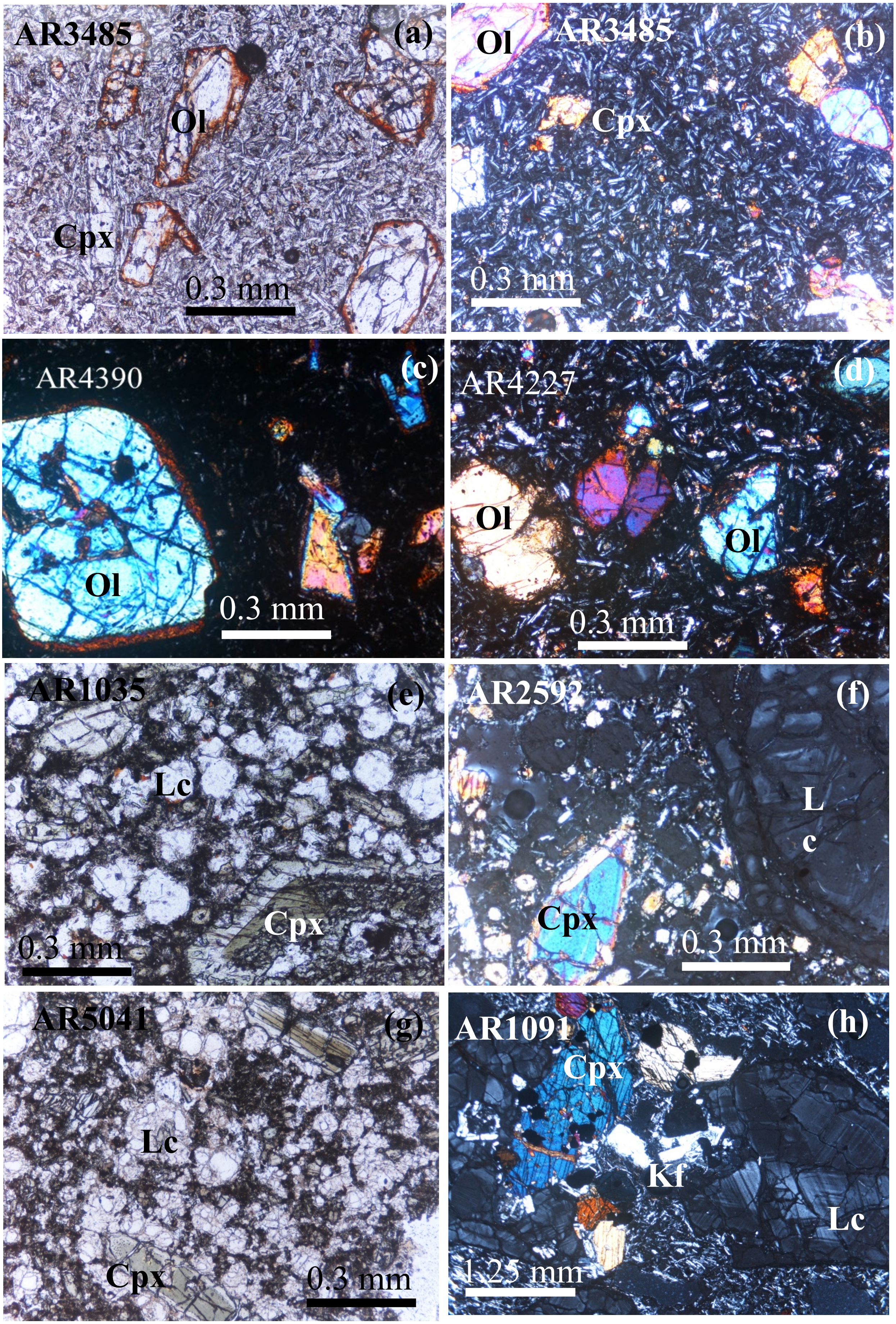

AR3485
(a)
Ol
Cpx
0.3 mm
Ol
AR3485
(b)
Cpx
0.3 mm
AR4390
(c)
Ol
0.3 mm
AR4227
(d)
Ol
Ol
0.3 mm
AR1035
(e)
Lc
Cpx
0.3 mm
AR2592
(f)
L
c
Cpx
0.3 mm
AR5041
(g)
Lc
Cpx
0.3 mm
AR1091
(h)
Cpx
Kf
Lc
1.25 mm

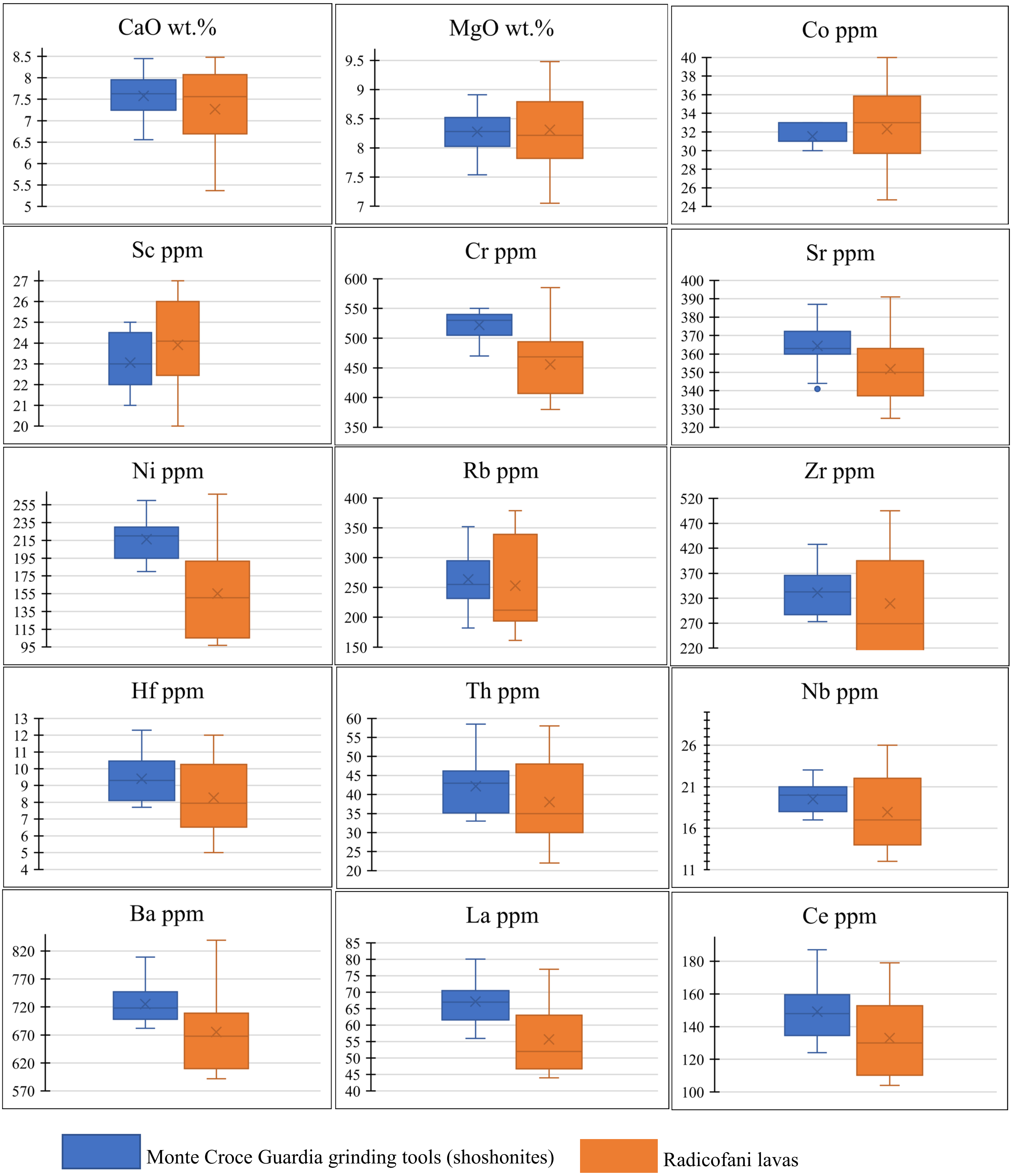
CaO wt.%
MgO wt.%
Co ppm
Sc ppm
Cr ppm
Sr ppm
Ni ppm
Rb ppm
Zr ppm
Hf ppm
Th ppm
Nb ppm
Ba ppm
La ppm
Ce ppm
Monte Croce Guardia grinding tools (shoshonites)
Radicofani lavas

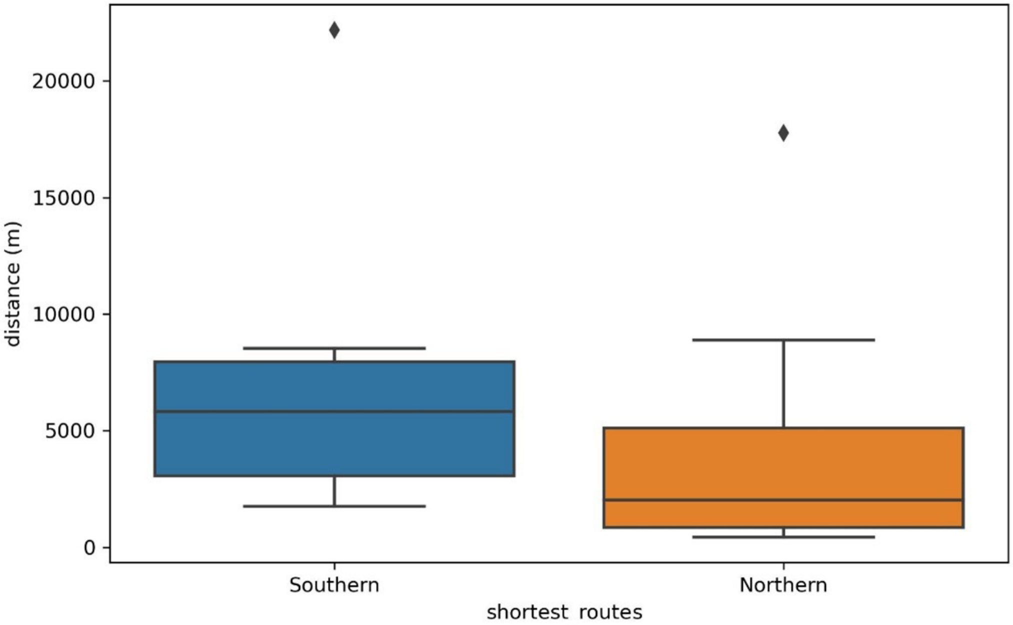

20000
15000
10000
5000
0
distance (m)
Southern
Northern
shortest routes

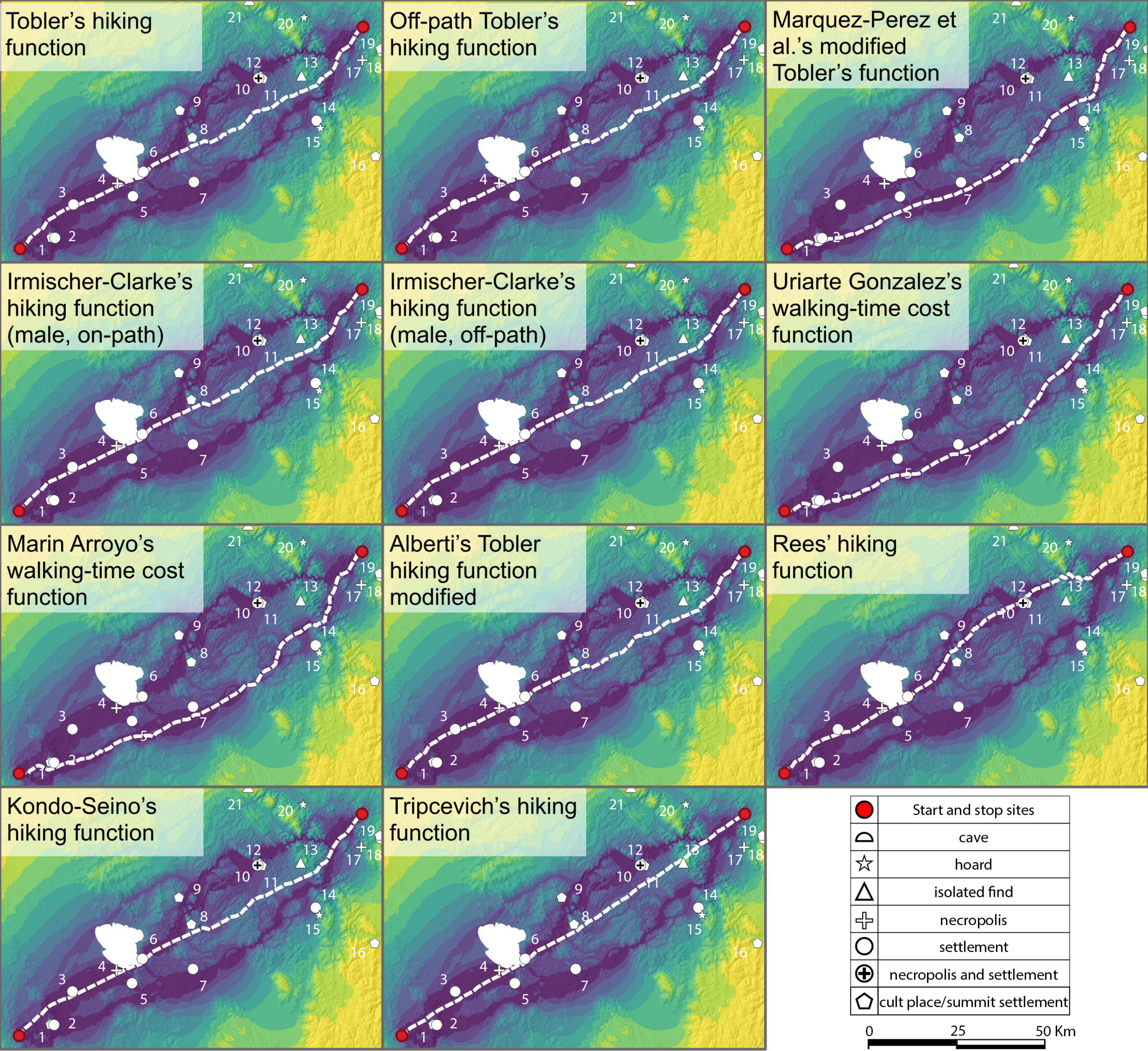

Tobler's hiking function
Off-path Tobler's hiking function
Marquez-Perez et al.'s modified Tobler's function
Irmischer-Clarke's hiking function (male, on-path)
Irmischer-Clarke's hiking function (male, off-path)
Uriarte Gonzalez's walking-time cost function
Marin Arroyo's walking-time cost function
Alberti's Tobler hiking function modified
Rees' hiking function
Kondo-Seino's hiking function
Tripcevich's hiking function
Start and stop sites
cave
hoard
isolated find
necropolis
settlement
necropolis and settlement
cult place/summit settlement
0
25
50 Km

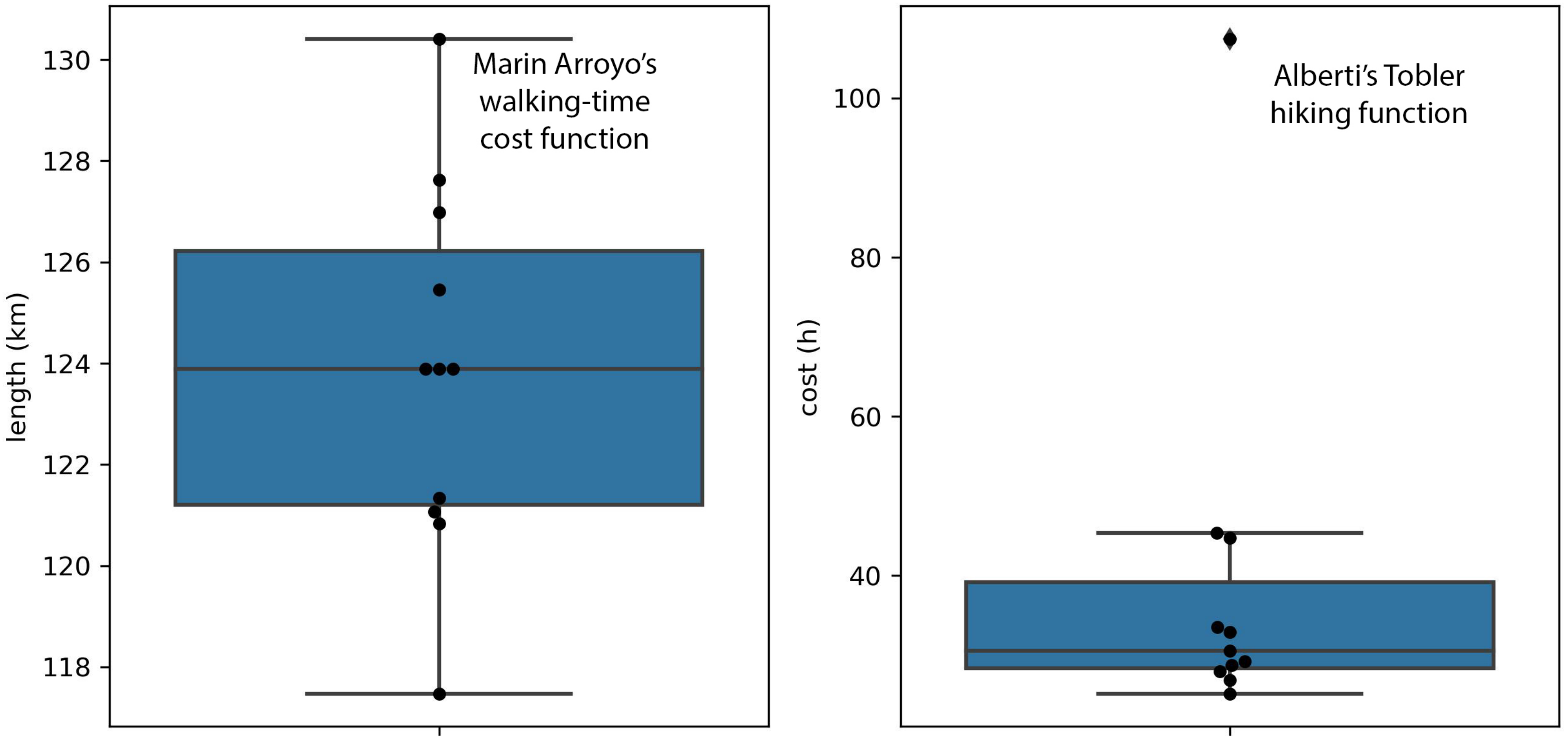

length (km)
130
128
126
124
122
120
118
Marin Arroyo's
walking-time
cost function
cost (h)
100
80
60
40
Alberti's Tobler
hiking function

High K-Series	
AR1091	Leucite phonolite
AR679	Leucite tephrite
AR1035	Leucite tephrite
AR 1981	Leucite tephrite
AR2592	Leucite tephrite
AR5041	Leucite tephrite
**K-Series**	
AR642	Shoshonite
AR814	Shoshonite
AR852	Shoshonite
AR863	Shoshonite
AR1020	Shoshonite
AR1113	Shoshonite
AR1202	Shoshonite
AR1649	Shoshonite
AR1826	Shoshonite
AR2259	Shoshonite
AR2535	Shoshonite
AR3286	Shoshonite
AR3485	Shoshonite
AR4009	Shoshonite
AR4227	Shoshonite
AR4390	Shoshonite
AR4540	Shoshonite

		Leucite phonolite			Leucite tephrites										Shoshonites									
**wt.%**	D.L	**AR1091**	**AR679**	**AR1035**	**AR1981**	**AR2592**	**AR5041**	**AR642**	**AR814**	**AR852**	**AR863**	**AR1020**	**AR1113**	**AR1202**	**AR1649**	**AR1826**	**AR2259**	**AR2535**	**AR3286**	**AR3485**	**AR4009**	**AR4227**	**AR4390**	**AR4540**
SiO_2	0.01	53.85	46.57	48.1	43.19	46.13	47.89	52.56	54.27	52.97	54.29	54.5	53.63	52.27	52.75	52.76	52.05	54.42	53.27	53.29	54.65	52.67	55.42	53.08
Al_2O_3	0.01	20.45	17.77	17.63	16.41	17.51	17.12	15.44	14.27	15.58	14.98	15.01	15.01	15.95	15.8	15.74	14.89	14.47	15.49	14.6	15.35	15.9	14.66	15.2
Fe_2O_3t	0.01	4.24	7.62	7.66	8.39	7.58	8.72	6.88	6.47	6.91	6.88	6.88	7.01	7.51	7.06	7.34	7.11	6.42	6.78	6.78	6.77	7.34	6.58	6.81
MnO	0.001	0.14	0.17	0.16	0.17	0.16	0.17	0.14	0.10	0.11	0.10	0.12	0.11	0.11	0.11	0.12	0.12	0.10	0.12	0.11	0.11	0.10	0.10	0.11
MgO	0.01	0.72	4.01	3.92	4.8	4.14	4.03	7.99	8.91	8.16	8.5	8.23	8.54	7.54	7.91	8.41	8.72	8.3	7.57	8.84	8.21	8.06	8.28	8.48
CaO	0.01	4.39	10.01	9.7	11.34	10.36	10.5	7.69	6.56	7.75	7.25	7.46	7.51	8.45	8.44	8.23	6.62	7.3	8.04	7.63	7.87	7.86	6.93	7.24
Na_2O	0.01	5.32	1.98	2.3	2.92	3.57	4.17	1.42	1.29	1.49	1.47	1.56	1.37	1.53	1.63	1.62	1.32	1.33	1.61	1.46	1.64	1.54	1.45	1.35
K_2O	0.01	4.57	5.03	5.45	3.67	1.85	3.08	4.37	5.03	3.91	4.39	4.19	4.62	3.71	3.61	3.54	3.98	5.08	3.75	4.36	3.79	3.61	4.61	4.37
TiO_2	0.001	0.56	0.75	0.74	0.91	0.76	0.84	1.10	1.20	1.07	1.11	1.07	1.07	1.03	1.04	1.02	1.07	1.11	1.01	1.06	0.98	1.05	1.09	1.15
P_2O_5	0.01	0.45	0.51	0.54	1.06	0.98	0.5	0.44	0.52	0.38	0.4	0.38	0.44	0.4	0.38	0.35	0.4	0.44	0.41	0.4	0.33	0.38	0.37	0.41
LOI		4.93	5.33	3.95	7.35	7.06	3.09	2.36	1.62	1.4	1.06	0.83	1.18	1.69	1.69	1.24	2.58	1.12	1.81	1.67	0.78	1.9	1.19	2.03
Total		99.61	99.76	100.1	100.2	100.1	100.1	100.4	100.3	99.73	100.4	100.2	100.5	100.2	100.4	100.4	98.86	100.1	99.85	100.2	100.5	100.4	100.7	100.2
Alkali		9.89	7.01	7.75	6.59	5.42	7.25	5.79	6.32	5.4	5.86	5.75	5.99	5.24	5.24	5.16	5.3	6.41	5.36	5.82	5.43	5.15	6.06	5.72
**ppm**																								
Sc	1	1	16	15	20	16	15	23	21	24	22	23	22	25	24	25	23	21	25	22	24	25	21	22
V	5	135	245	252	224	245	299	167	152	172	163	165	161	177	176	178	167	160	179	164	174	178	154	165
Cr	20	< 20	60	50	60	60	< 20	530	550	530	540	470	550	510	490	540	530	540	510	530	500	530	500	530
Co	1	6	23	23	27	23	26	31	31	31	33	33	33	30	31	32	33	32	31	33	30	30	31	31
Ni	20	< 20	60	50	70	60	50	210	260	200	230	220	230	180	200	190	230	250	180	230	190	200	230	250
Rb	2	272	465	531	362	335	418	255	352	253	303	287	277	188	240	201	250	348	223	274	247	182	324	279
Sr	2	2489	2484	2353	939	2424	2071	383	387	360	365	368	360	360	366	360	305	373	344	361	341	347	370	385
Y	1	44	36	36	30	36	35	22	21	21	21	21	20	22	21	22	24	21	24	23	20	21	20	23
Zr	2	816	449	425	244	356	387	333	428	321	358	331	340	273	288	278	333	383	277	348	286	299	374	377
Nb	1	61	27	28	29	25	21	20	23	20	21	19	20	17	18	17	20	22	18	19	17	18	21	22
Ba	2	2493	1809	1845	910	1916	1786	745	762	698	698	713	709	723	692	682	749	725	703	718	686	772	745	809
La	0.1	243	156	160	106	156	132	66.9	80.1	65.6	69.2	64.6	67	58.3	59.5	58.5	70.5	74.1	61.9	69.8	56	61.6	70.2	75.9
Ce	0.1	427	288	292	203	289	258	148	187	143	157	148	153	124	137	129	150	175	133	157	126	136	162	170
Pr	0.05	41	32	32.5	23.6	33.3	29.7	19.4	25	18.4	20	19.1	19.6	15.8	17.2	16.1	20	22.5	16.8	20.1	15.8	17.5	20.4	22.4
Nd	0.1	131	114	118	88.5	122	113	77.4	101	74.6	80.4	75.6	78.1	62.6	67.6	63.7	80.4	87.4	66.1	79.8	63.5	69.9	81.9	90.6
Sm	0.1	18.8	19.1	20.3	14.6	21	19.8	12.5	15.1	11.7	12.7	11.6	12.5	10.3	10.6	10.3	12.6	13.8	10.8	12.2	10.1	10.7	12.6	13.5
Eu	0.05	3.58	3.61	4.11	2.95	4.17	3.92	2.29	2.51	2.17	2.25	2.16	2.27	1.97	2.05	1.98	2.28	2.37	2.1	2.27	1.96	2.07	2.21	2.4
Gd	0.1	11.7	13.6	13.5	10.1	13.9	13.1	7.3	7.8	6.9	7.1	6.8	7	6.5	6.6	6.2	7.5	7.3	6.7	7.2	6.3	6.7	6.9	7.6
Tb	0.1	1.7	1.7	1.6	1.3	1.7	1.5	0.9	0.9	0.9	0.9	0.9	0.9	0.8	0.8	0.8	0.9	0.9	0.9	0.9	0.8	0.8	0.8	0.9
Dy	0.1	8.2	8	8.1	6.5	8.3	7.8	4.8	4.7	4.7	4.7	4.3	4.5	4.5	4.6	4.5	4.9	4.6	4.8	4.7	4.1	4.4	4.2	4.8
Ho	0.1	1.5	1.4	1.4	1.1	1.4	1.3	0.9	0.8	0.9	0.9	0.8	0.9	0.8	0.8	0.8	0.9	0.8	0.9	0.9	0.8	0.8	0.8	0.9
Er	0.1	4.4	3.8	3.5	3	3.6	3.3	2.4	2.3	2.4	2.3	2.2	2.3	2.4	2.3	2.4	2.4	2.3	2.6	2.4	2.2	2.3	2.2	2.4
Tm	0.05	0.62	0.48	0.47	0.41	0.47	0.41	0.34	0.3	0.35	0.33	0.31	0.31	0.32	0.31	0.32	0.34	0.31	0.35	0.31	0.3	0.33	0.3	0.33
Yb	0.1	3.9	2.8	2.9	2.5	2.8	2.5	2.1	2	2.1	2.1	2.1	2	2.1	2	2	2.2	1.9	2.3	2.1	1.9	2.1	1.9	2.2
Lu	0.01	0.64	0.4	0.43	0.35	0.43	0.39	0.33	0.31	0.34	0.31	0.31	0.32	0.33	0.32	0.32	0.33	0.3	0.35	0.31	0.31	0.32	0.28	0.31
Hf	0.2	12.8	8.8	9.2	5	5.7	8.3	9.3	12.3	9.2	10.4	9.4	10.1	7.7	8.3	7.9	9.6	11	7.8	9.3	7.8	8.3	10.5	11
Ta	0.1	2.8	0.9	1.2	1.4	1.1	0.9	1.4	1.6	1.2	1.5	1.3	1.4	1.1	1.2	1.1	1.4	1.5	1.2	1.3	1.4	1.1	1.4	1.7
Th	0.1	179	78.4	80.9	44.4	77.2	62.9	43	58.5	40.8	46.4	43	44.1	33.7	37.3	34.5	43.1	50.5	34.9	43.9	33	35.4	45.9	48.9
U	0.1	40.3	14.4	15.8	4.2	16.7	13.4	7.1	10.1	7	8.4	7.8	7.5	6	6.7	5.9	6.7	9.3	6.3	7.1	6.3	6.4	8.4	8.1

D.L. = Detection Limit.

**Functions**	**Length (km)**	**Cost (h)**
Tobler's hiking function	123,89	26,86
Off-path Tobler's hiking function	123,89	44,76
Marquez-Perez et al.'s modified Tobler's function	127,62	33,52
Irmischer-Clarke's hiking function (male, on-path)	121,34	32,90
Irmischer-Clarke's hiking function (male, off-path)	120,83	45,33
Uriarte Gonzalez's walking-time cost function	125,45	25,15
Marin Arroyo's walking-time cost function	130,41	27,95
Alberti's Tobler hiking function modified for pastoral foraging excursions	123,89	107,43
Rees' hiking function	126,98	28,72
Kondo-Seino's hiking function	121,07	29,24
Tripcevich's hiking function	117,47	30,54
