# Supplementary material for: Tracing the human movements of three thousand years ago by volcanic grinding tools in the Final Bronze Age settlement of Monte Croce Guardia (Arcevia-Marche Region, central Italy)
Source: Sci Rep. 2023 Apr 29;13:7022. doi: 10.1038/s41598-023-34033-x (PMC10148880; doi:10.1038/s41598-023-34033-x)
Supplement: Supplementary file 2 — Supplementary Information 2. [file 41598_2023_34033_MOESM2_ESM.docx]

**Supplementary Material**

Fig. S1 - Top: plans of the huts excavated during the 2015-2021 campaigns; bottom: map showing the areas and the chronology of the archaeological excavation since 1961.

Fig. S2 - Thin section petrography of representative fragments of volcanic grinding tools by optical polarized microscope at plane polarized light (a, e, g) and crossed Nicols (b, c, d, f, h).

Fig. S3 - Box-plot diagrams showing the main composition of the studied fragments of grinding tools compared with volcanic rocks from Radicofani volcanic center^68^. Coloured boxes represent the standard 50% of the data whereas the range values are defined by vertical bars (excluding outlier samples as single points). The median and average values are respectively marked by horizontal line and a cross inside the boxes. The wide range values of the Radicofani volcanic rocks is mostly due to their lithological and chemical variability.

Fig. S4 - Box-plot diagram summarizing the distance between the considered archaeological contexts/sites reported in Fig. 7 and the two pathways joining Radicofani and Monte Croce Guardia. The median distance between the sites and the Northern route is 2 km, while it increases to 5 km in the case of the Southern route.

Fig. S5 - Maps showing the most convenient paths from Radicofani to Monte Croce Guardia according to 11 different cost functions used (see Table S3 for details) according to the Movecost R-package^8^. Numbers refer to the different contexts of the Final Bronze Age archaeological sites: 1 Monte Cetona (summit); 2 Case Carletti; 3 Chiusi; 4 Panicarola; 5 Monte Solare; 6 San Savino; 7 Perugia - Via Settevalli; 8 Monte Tezio; 9 Monte Acuto; 10 Gubbio - Via dei Consoli e Vescovado; 11 Monte Ansciano; 12 Monte Ingino; 13 Costacciaro; 14 Colle Mori; 15 Gualdo Tadino - hoard; 16 Monte Primo; 17 Pianello di Genga; 18 Gola della Rossa; 19 Monte Murano; 20 Frontone - Chiuse; 21 Fondarca - Grotta delle Nottole.

Fig. S6 - Box-plot diagram summarizing the results of the application of 11 cost functions^8^ to define the most convenient path from Radicofani to Monte Croce Guardia. Left: distribution of path lengths expressed in kilometers (km); rigth distribution of walking-time expressed in hours (h).

Table S1 - Summary of samples collected in the archaeological site of Monte Croce Guardia and their relative composition (classification and magmatic series).

Table S2 - Whole-rock major oxides and trace elements composition of the studied fragments of volcanic grinding tools.

Table S3 - Functions used to define the most convenient path from Radicofani to Monte Croce Guardia according to the R-package Movecost^8^.
